# From Sensory Perception to Lexical-Semantic Processing: An ERP Study in Non-Verbal Children with Autism

**DOI:** 10.1371/journal.pone.0161637

**Published:** 2016-08-25

**Authors:** Chiara Cantiani, Naseem A. Choudhury, Yan H. Yu, Valerie L. Shafer, Richard G. Schwartz, April A. Benasich

**Affiliations:** 1 Center for Molecular and Behavioral Neuroscience, Rutgers University, Newark, New Jersey, United States of America; 2 Child Psychopathology Unit, Scientific Institute IRCCS Eugenio Medea, Bosisio Parini, Lecco, Italy; 3 Psychology, SSHS, Ramapo College of New Jersey, Mahwah, New Jersey, United States of America; 4 The Graduate Center, City University of New York, New York, New York, United States of America; 5 Department of Communication Sciences and Disorders, St. John's University, New York, New York, United States of America; Vanderbilt University, UNITED STATES

## Abstract

This study examines electrocortical activity associated with visual and auditory sensory perception and lexical-semantic processing in nonverbal (NV) or minimally-verbal (MV) children with Autism Spectrum Disorder (ASD). Currently, there is no agreement on whether these children comprehend incoming linguistic information and whether their perception is comparable to that of typically developing children. Event-related potentials (ERPs) of 10 NV/MV children with ASD and 10 neurotypical children were recorded during a picture-word matching paradigm. Atypical ERP responses were evident at all levels of processing in children with ASD. Basic perceptual processing was delayed in both visual and auditory domains but overall was similar in amplitude to typically-developing children. However, significant differences between groups were found at the lexical-semantic level, suggesting more atypical higher-order processes. The results suggest that although basic perception is relatively preserved in NV/MV children with ASD, higher levels of processing, including lexical- semantic functions, are impaired. The use of passive ERP paradigms that do not require active participant response shows significant potential for assessment of non-compliant populations such as NV/MV children with ASD.

## Introduction

Autism spectrum disorders (ASD) are complex neurodevelopmental conditions, characterized by a range of difficulties including impairments in social cognition, deficits and delays in language and communication abilities and restricted interests or activities, as expressed by repetitive patterns of behavior [1]. The impairments in language and communication may be present to a greater or lesser degree, depending on the severity of the disorder, IQ, and age of the individual [2]. There is tremendous variability in the expression of such impairments—which range from the complete absence of functional expressive language (mutism) to subtle anomalies in the pragmatic aspects of language. However, the systematic study of language has been almost exclusively limited to those children with ASD who have acquired some functional language, whereas the minimally-verbal end of the spectrum has been seriously neglected, as recently reported by Tager-Flusberg and Kasari [3]. Even so, it has been suggested that the linguistic skills of children diagnosed as ASD might well be underestimated due to the demand characteristics of language tests and the testing situation [4]. More specifically, standardized testing is essentially a one-to-one social situation; thus successful performance on such tasks requires functional communication skills as well as competency in social interaction. Since both these domains may be affected in individuals with ASD, it is difficult to isolate the source of an apparent failure [5]. Other techniques such as analysis of spontaneous or elicited speech samples are also considered problematic for studies with ASD populations, as it is difficult to engage these children in a social situation [6]. Taken together, these challenges point to the necessity of developing different approaches to the assessment of linguistic and cognitive abilities in ASD populations that do not confound linguistic performance with difficulties meeting task demands. This is particularly important when investigating the existence and level of language skills in children with ASD who are essentially nonverbal. Nonverbal (NV) or minimally verbal (MV) populations are frequently labeled "low-functioning" even when little or no traditional data can be gathered as to cognitive or language level. This label cannot easily be challenged given the very few studies that include children in this group. In addition, many researchers have avoided testing this group using methods such as electroencephalography (EEG), because they believed that it would be too difficult to keep these children calm and attentive within the experimental situation long enough to acquire even passive EEG data. Questions remain about whether NV/MV ASD children comprehend any incoming linguistic input, their functional level of receptive language and whether the speech that *is* perceived is processed in a manner comparable to that of the typically developing child. Skwerer and colleagues [4] recently compared several adapted measurement tools to assess receptive language ability in minimally verbal children and adolescent with ASD. Their measures included a standardized direct assessment of receptive vocabulary, a caregiver report measure, an eye-tracking test of word comprehension and a computerized assessment using a touch screen. The results were highly variable across all measures, with eye tracking and computerized assessment approaches providing more reliable assessment of comprehension than the commonly used standardized tests. However, the authors conclude by suggesting that more research is needed before any of these innovative, technology-based assessment tools are ready for integration into clinical practice.

### Event-Related Potentials

Event-Related Potentials (ERPs) derived from electroencephalography (EEG) allow unique insight into the study of language in nonverbal populations. One of the main advantages of ERPs is that the electrophysiological response to cognitive stimuli, including linguistic stimuli such as words or sentences, can be recorded in the absence of any overt behavioral response and can thus be used to infer stimulus encoding, discrimination, and higher-level cognitive processes. Multiple paradigms have been developed to assess language comprehension with ERPs at the semantic, syntactic and pragmatic levels, and these designs have been successfully implemented in prelinguistic infants (for a review see [7]), non-verbal children (e.g., children with severe congenital brain disorders such as cerebral palsy and holoprosencephaly, see [8,9]), and children and adults with mild-to-moderate to severe language disorders (e.g., [10], for a review see [11]). As such, ERPs can provide an essential tool for understanding linguistic skills in the nonverbal ASD population. Importantly, ERPs also provide detailed temporal information about the processing of incoming linguistic stimuli, thus allowing investigation of the time-course of processing in the tens-of-milliseconds range for visual and/or auditory stimuli that carry linguistic information. Thus, both sensory/perceptual and higher-order linguistic skills can be examined in order to delineate the link between these two levels of processing.

### Language Development: From sensory perception to lexical-semantic processing

Research on language development has demonstrated dependencies between lower-order sensory perception and higher-order linguistic skills. Basic perceptual processing skills constrain the acquisition of higher level-linguistic processing, and in turn, higher-level processes (e.g., acquisition of word meaning) modulate lower levels of processing. For example, fine-grained basic auditory skills during infancy are prerequisites to decoding the speech stream [12–14]. Auditory-perceptual skills allow the developing brain to build acoustic-phonetic maps that represent the sounds of its native language [15] and via a cascading process, these skills impact the development of the lexical forms. In turn, as words are added to the lexicon, they bring about reorganization of the acoustic-phonetic relationships within the lexical network, resulting in a phonological system that influences speech perception [16].

Another ability that underlies the development of essential linguistic and categorization skills is the ability to process cross-modal input, as words occur in the auditory modality, whereas objects and other referents are experienced visually. Much of the early stages of word learning consist of mapping auditory forms to visual objects [17]. Thus, the ability to process and allocate attention to visual stimuli also makes an important contribution to language acquisition. In this sense, auditory and visual sensory/perceptual abilities and higher-order linguistic skills can be considered as tightly interrelated systems, where deficits in basic processing skills will be reflected in higher-order skills and deficits in higher-order skills can impact basic lower-order skills.

Since it is unknown whether and at which levels linguistic skills are impaired in NV/MV children with ASD, this study is intended to investigate both basic sensory/perceptual abilities and higher-order linguistic skills in this population using an ERP paradigm based on a picture-word matching task (see [8] for the use of a similar paradigm in children with cerebral palsy). In these paradigms, children are passively presented with a picture followed by an auditory label that either matches the picture (match condition) or does not (mismatch condition). This paradigm typically elicits basic visual ERP peaks in response to the picture (i.e., P1 and PSW), basic auditory ERP peaks in response to the auditory word label (i.e., P1) and finally a negativity occurring between 200 and 700 ms (i.e., N400) after presentation of the word, specifically when it is semantically incongruent with respect to the previous picture [18]. In the next sections, the literature on ERP evidence of sensory/perceptual and high order linguistic processing in typically developing populations and specifically in ASD adults and children will be reviewed.

### Basic perceptual processing of visual and auditory stimuli

Basic sensory processes, examined via obligatory ERP components, have been frequently investigated in children and adults with ASD. In typically developing populations, the most readily identifiable obligatory visual ERP component is the P1 peak. It begins at about 70–90 ms, peaking at around 80–130 ms in adults and older children, but at later latencies in young children. P1 has maximum amplitudes over lateral occipital sites [19] and has been shown to be modulated by attention [20–22]. Attention allocation to the correct/target area results in greater P1 amplitude while attention allocation to the *incorrect* location results in a decrement in P1 amplitude [23]. The decrement or suppression of the P1 may represent the cost of having to stop attending to one area and then shifting attention to the location of the target stimulus. In addition, late slow wave activity has been reported in many studies as a response to visual or auditory stimuli (see [24], for a review). This late activity (often called a positive slow wave: PSW) usually emerges after 300 ms, whenever target detection leads to a complex subsidiary task. It reflects a general, or diffuse, activation of neural systems and is typically characterized by the absence of an identifiable peak and a slow return to baseline levels [25]. The PSW component has been described as a reflection of the extent of stimulus encoding and updating in working memory (e.g., [24]). In the present study, the emergence of a PSW was expected in response to the visual presentation of a meaningful picture, as an index of the automatic activation of memory traces related to that picture (if it is familiar) and of the effort to retrieve a label for it.

Individuals with ASD may have basic visual processing impairments (e.g., [26–28]). High functioning ASD participants show abnormal latency, amplitude and morphology for the obligatory visual peak (visual P1) (e.g., [26–28]), as well as components tapping into higher-level processing (e.g., P300) (e.g., [29,30]). These findings corroborate behavioral results suggesting that high-functioning ASD individuals have atypical sensory processing abilities with regard to complex (but not simple) stimuli, distorted selective and sustained attention abilities as well as altered inhibitory control that interferes with the ability to extract meaning from an ongoing stream of information (see [31], for a review). In a broader context, these findings are in line with a detail-focused processing deficit (referred to as “weak central coherence” [32]), that has been described in individuals with ASD. This deficit is characterized by enhanced attention to local details and a consequent failure to extract the gestalt of the input. This early visual processing impairment has been hypothesized to account for the face processing deficits often seen in ASD, and thus might contribute to higher-level social and cognitive difficulties [33].

Evidence for abnormalities in basic auditory processing in individuals with ASD is still inconsistent and seems to depend on the stimulus type used (non-speech tones or verbal stimuli) (see [34], for a review). Throughout the early school years, children’s obligatory auditory ERPs are dominated by P100, N250 and N450 peaks, although with slow presentation rates, an adult-like P1-N1-P2 pattern can be observed [35]. As described by Ceponiene et al., important developmental changes emerge from 4 to 9 years of age. The auditory P1 peak is described as a modality-specific component that is generated within the auditory cortex and reflects early perceptual processes associated with the detection of an acoustical event [36]. It is reported to peak approximately 50 ms after stimulus onset in adults and 100 ms after stimulus onset in children [37]. The N1 peak is thought to be generated in the supra-temporal auditory cortex and to show the most extensive developmental changes [35,38]. The N1 peak (specifically, N1b) can be observed in children at slower rates of presentation (> 1 sec), peaking between 100 and 200 ms, but is attenuated in children at fairly fast stimulus presentation rates (< 1 sec) [39].

Previous studies comparing children with ASD and typically developing children on the obligatory auditory components have reported highly inconsistent results. Two studies conducted with low functioning children with ASD found shorter latencies [40,41]. Some studies of higher-functioning children with ASD have found no differences between groups [42–45] and others have observed longer latencies [30,46–49]. Dunn and colleagues investigated obligatory components in response to real words in children with ASD. Their findings suggest significantly delayed obligatory auditory peaks in high-functioning children with ASD as compared to their typically developing peers [50,51]. The shorter latencies previously reported in the auditory obligatory components of ASD groups may reflect intact processing of basic auditory stimuli such as tones or consonant-vowel syllables e.g. /da/ or /ga/, but impaired processing of meaningful stimuli such as words or sentences. Such findings imply intact processing in primary auditory cortex in individuals with ASD but compromised processing along higher-order pathways, possibly involving projections to hippocampus, prefrontal, frontal and parietal cortex [52].

### High-order linguistic processing (lexical-semantic integration)

The N400 component is a negativity occurring between 200 and 700 ms after presentation of a stimulus that is semantically incongruent with the previous context [18]. It is thought to represent lexical-semantic processing [53–56]. In semantic priming paradigms, the amplitude of the N400 increases in response to targets (words or pictures) that do not match the semantic expectation built up by the previously presented primes (words or pictures) [57]. A number of developmental studies have investigated the N400 component in toddlers and children, both in sentential contexts [58–62] and in semantic priming paradigms [8,63–67]. Studies that have used the semantically-incongruent word paradigm, have shown the presence of the N400 in participants as young as 19 months of age [65]. Unlike the mature N400 component, however, in children the effect is usually longer and more frontally distributed [7].

A few studies have used the N400 and the semantic priming paradigm to investigate language abilities in higher-functioning ASD children [50,51,68,69]. In one of the first such studies, eight high-functioning ASD children (7 to 10 years of age) and eight aged-matched neurotypical children were auditorily presented with words belonging to a specific semantic category (animals) and words that were considered to be out-of-category [51]. Children were asked to respond with a finger lift to words that were in-category (animals) and to inhibit responding to words that were out-of-category. Behavioral performance suggested that the group with ASD was significantly slower to respond to the targets than the control group. No statistically significant difference was seen in error rates between ASD and typically developing children; however, the means were in the expected direction favoring typically developing children. The ERP results were more revealing in that children with ASD as well as controls showed a clear negative peak for both conditions; however, this negativity was more robust for out-of-category words for the control children, consistent with N400 modulation, while no difference was observed for children with ASD. These results have been replicated with a larger sample that included older children (18 high-functioning ASD children, 10 aged 8–9 years and eight aged 11–12 years) [50].

In a similar experimental paradigm, adolescents with ASD and typically developing adolescents were presented with pictures of animals (e.g., “dog”) and animal sounds (e.g., “woof”). Pairs of pictures and sounds could be congruent (e.g., “dog” paired with “woof”) or incongruent (e.g., “dog” paired with “ribbit”) and were presented either simultaneously or sequentially [70]. The results of the study by Russo et al. [70] showed that, within this specific context, adolescents with ASD appeared to process semantic incongruency. In particular, control participants exhibited responses to incongruence in the expected latency range of 300–500 ms (N400) with maximal activation at frontal channels. In the participants with ASD the modulation of incongruency was still present but occurred at earlier latencies (between 150 and 300 ms), more reflective of perceptual rather than higher-order cognitive processes, while recruiting posterior and centro-temporal scalp regions [70]. These findings contrast with previous evidence of absent or reduced N400 among ASD subjects [50,51,68,69]. Finally, in a recent study of high functioning children with ASD (4 to 7 years of age), McCleery et al. [69] paired pictures with matched or mismatched word labels (e.g. a picture of a car with either the word “car” or the word “ball”), or with matched and mismatched environmental sounds (e.g., a picture of a car with either the sound of a car engine starting or the sound of ball bouncing). While similar N400 morphology was seen for children with ASD and typically developing children in the environmental sound condition, no N400 was evident in the ASD group for the word condition. However, there is no way of knowing if a subset of the children in the ASD group did indeed exhibit the N400. One of the limitations of the literature reviewed here is that individual results are never reported. To our knowledge, similar processes have not been studied with NV/MV children with ASD. Since the population of NV/MV children with ASD is rarely studied and is unique and likely heterogeneous, their electrophysiological profile is difficult to predict from previous studies of ASD that included individuals who were substantially higher functioning in language and general cognitive abilities. Moreover, this difficulty in comparing ASD study populations is compounded by the variations in the stimuli and paradigms employed.

The purpose of the present investigation was to use EEG/ERPs to examine the time course of sensory/perceptual and higher-order linguistic skills in NV/MV children with ASD, and to compare it with that of typically-developing children. The specific paradigm used in the study allowed us to investigate the entire pattern of information processing, from very early sensory perception (for both visual and auditory processing) all the way through to lexical-semantic processing. To our knowledge, no studies to date have examined the neural correlates of higher-level linguistic processing in this population. The presence/absence of these electrophysiological components in a group of NV/MV children diagnosed as ASD can provide insight into their comprehension skills, receptive vocabulary and lexical-semantic processing. In addition, analysis of the obligatory components elicited by visual presentation of pictures and auditory presentation of words should provide information about lower-level sensory processes and about the integration of visual and auditory information in these children.

## Materials and Methods

### Participants

Fifteen NV/MV children with ASD aged between 3 years, 7 months and 7 years, 11 months were recruited for the study through the NJ Autism and Language Genetics Study (NJLAGS), the International Autism Network (IAN) and private schools for children with Autism in the metropolitan New York/New Jersey area. Prior to scheduling the ERP experiment, the children participated in training sessions based on desensitization and traditional behavior modification techniques to facilitate wearing a 64-channel sensor net (see [71]). These training sessions took place at children’s houses and required at least two researchers to visit the home. Generally, one to six home visits were needed to train the children to wear the net (on average, 2.6 training sessions for each child). Eighty percent (80%) of the children with ASD (n = 11) were successfully trained to wear the net and were able to participate in the ERP experiment. However, one child was excluded due to excessive movement during the EEG recording. The final sample consisted of 10 children with ASD (three females) between 4 years, 5 months and 7 years, 4 months of age (*M* = 6.28; *SD* = 1.14). All children with ASD included in the final sample were diagnosed with an Autism Spectrum Disorder by a developmental pediatrician, neurologist, or a licensed clinical psychologist prior to recruitment. This sample also met the following criteria: (a) product of a full-term pregnancy, (b) no history of head trauma or other neurological conditions (e.g., seizures) or comorbid genetic conditions (e.g., Fragile X), c) English as the primary language spoken in the home. All participants were enrolled in self-contained special education classes for children with ASD and were receiving individualized education programs (e.g., Applied Behavioral Analysis, ABA). In addition, all received speech/language therapy and occupational therapy with an emphasis on sensory integration. An intake questionnaire created for this study was administered to the parents to collect more detailed information and to exclude the presence of any additional diagnoses (e.g., Tuberous sclerosis, fragile X, Down Syndrome). As reported in Table 1, all children with ASD had an expressive vocabulary of less than five functional/intelligible words used on a daily basis, except one 7-year-old child with ASD who reportedly had an expressive vocabulary of around 20 words. Only two children used phrases of at least 2–3 words that include a verb, but most of the parents reported that their children understand simple verbal directions and overall to understand “more than he/she expressed” (n = 9).

**Table 1:** Summary of communication abilities.

ID	Child speaks	Expressive vocabulary (# Words)	Phrases	Follows 1-step command	Seems to understand more than expresses	Signs (single)	Assisted communi-cation (PECS)	Electronic Devise
ASD1	Y	20	Y	Y	Y	N	Y	Y
ASD2	N	0	N	S	Y	Y	Y	N
ASD3	N	0	N	S	Y	Y	N	N
ASD4	S	2	N	N	Y	Y	Y	N
ASD5	N	0	N	Y	Y	Y	N	N
ASD6	S	4	N	Y	Y	Y	N	N
ASD7	S	3	N	S	Y	N	N	N
ASD8	Y	3	N	S	Y	Y	Y	N
ASD9	S	5	Y	S	Y	Y	Y	N
ASD10	N	0	N	S	S	Y	Y	Y

Y = Yes.

S = Sometimes.

N = N.

Overall, the qualitative information collected from parental reports is summarized in Table 1. All but one of the participants with ASD exhibited language comprehension at or beyond the 16-month level (understand simple directions “some of the time”, used some gestures and produced a few words). As described in the introduction, standardized testing is extremely difficult for these children, and when it is feasible with an adapted format, the results are often not reliable [5]. For these reasons, no standardized measures of IQ or hearing are available for this group. The presence of serious auditory difficulties (hearing loss) was however an exclusion criterion for all children based on parental and clinical reports.

Ten typically developing children (three females) were individually matched to children with ASD for gender and chronological age within 6 months. Ages ranged between 4 years, 1 month and 7 years, 8 months (*M* = 6.33; *SD* = 1.43). Neurotypical children were recruited from the metropolitan New York/New Jersey area. None of the neurotypical children had a history of receiving special education services. According to parent report, all children in the Neurotypical group were performing at least within the average range across various areas of cognition, including language development, speech/articulation, general learning, attention, social/emotional development, hearing and vision. In addition, they scored in the average to high-average range on a test of non-verbal IQ (Kaufman Brief Intelligence Test, K-BIT or Stanford-Binet Intelligence Scales) (K-BIT: data for five children, *M* = 116.2; *SD* = 11.24; Standford-Binet: data for three children, *M* = 118; *SD* = 7.81) and language (Peabody Picture Vocabulary Test, Fourth Edition [PPVT-4]—Preschool Language Scale, Fourth Edition [PLS-4] or Clinical Evaluation of Language Fundamentals, Fourth Edition [CELF-4]) (PPVT-4: data for three children, *M* = 116.67; *SD* = 14.50; PLS-4: data for three children, *M* = 122.67; *SD* = 4.93; CELF-4: data for four children, *M* = 111.25; *SD* = 11.29) all administered by a speech-language pathologist or a psychologist. All neurotypical children also passed a hearing screening at 500 Hz at 30 dB HL and 1000, 2000, and 4000 Hz at 20 dB HL. Other inclusion criteria for the neurotypical children were: (a) Full term pregnancy, (b) No known sensory impairment, history of head trauma, other neurological conditions (e.g., seizures, cerebral palsy,) genetic or co-morbid conditions (e.g., Fragile X), (c) English as the primary language spoken in the home.

All parents or caregivers gave written consent for their children to participate in and be video-recorded for the EEG/ERP study. All study procedures were approved by the Rutgers University and City University of New York (CUNY) Institutional Review Boards and were performed in accordance with the ethical standards laid down in the 1964 Declaration of Helsinki.

### Stimuli

The study employed a picture-word matching paradigm to assess electrophysiological brain activity related to lower-level visual and auditory processing and semantic integration. The stimulus word items in the picture-word matching task consisted of 60 basic-level nouns, selected from the "Words and Gestures” version of the MacArthur-Bates Communicative Development Inventories- 2nd edition (75%) and popular children’s picture books (25%) to ensure that they were likely to be highly familiar and easily comprehended by two-year old children. In addition, the pictures were selected based on a pilot study conducted on seven typically developing children between 3 and 9 years of age.

Visual stimuli were 60 full-color digitized photographs of animals (e.g., bird, chicken) or inanimate objects (e.g., bed, pencil) presented on a 54x30 cm Samsung monitor placed 81 cm from the participant. Pictures were presented in the middle of the computer monitor against a white background. Two images for each noun were used (e.g., two bird images). Only the pictures that were labeled consistently (100% accuracy) by typically-developing children in the pilot study were used in the experimental task. The same set of pictures was used for both the match and mismatch contexts; the same picture was repeated twice within the overall randomized order, once followed by a congruent word and once followed by an incongruent word.

Thirty words were selected to label the pictures, and an additional 30 words, not semantically or phonologically related to the pictures, were selected. All words were monosyllabic (e.g., *bird*, *bed*) or bisyllabic (e.g., *chicken*, *pencil*) and all were very high frequency words (the averaged occurrences / 1000,000 Words at 48 months, based on the online tool “ChildFreq”, designed to explore word frequencies in child language was M = 141, SD = 118 –[72]). For a complete list of the stimuli, see S1 Appendix. The words in the match and mismatch conditions were not the same, but did not differ with respect to word duration in milliseconds (matching words, *M* = 612.80, *SD* = 87.59; mismatching words, *M* = 634.47, *SD* = 82.66; *t*(58) = .985, *p* = .329).

The words were produced by a female native speaker of English and were digitally recorded at a sampling rate of 44.1 kHz (16 bit; mono). The recordings were performed in a sound-isolated room using a microphone. The speaker was instructed to produce the words in a neutral, naturally spoken manner. All stimuli were normalized to the same intensity and edited to remove silent spaces at the onset. Both the recording and the following editing were performed using Sound Forge 4.5 software (Sonic Foundry, 1996). Word stimuli varied in duration from 450 to 805 ms (M = 625.07, *SD* = 86.38). The words were presented auditorily at approximately 65 dB SPL via two loudspeakers situated 30 degrees to the right and left of midline, in front of the participant.

### Experimental procedure

**Fig 1:**
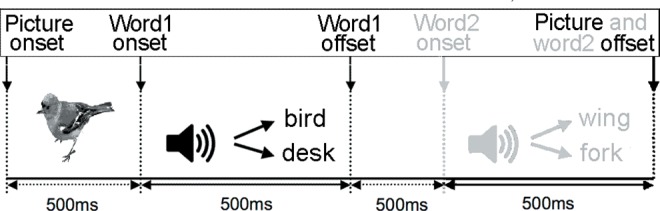
Sample trial. Sequence of events per trial, including timing, visual and auditory input. For the purpose of this study, only data from the picture and the first word were analyzed.

The resulting 120 items were arranged in four pseudo-randomized blocks, so that no more than five items of the same semantic properties (match vs. mismatch) were presented in a row. Each block contained 30 items, with a similar number of items per condition in each block. The picture and word stimuli were delivered by the E-prime software package (version 1.1). Fig 1 shows one exemplary trial. First, a fixation point consisting of a central cross was presented (500 ms in duration). Then still pictures of animals or inanimate objects were presented on the computer monitor for 2000 ms, corresponding to the total trial length. A word that either matched or mismatched the object/animal in the picture began after a 500-ms delay, while the picture remained on the screen. A second word, either semantically associated or non-associated with the first word was presented auditorily 500 ms after the offset of the first word. Here, we only report data from the picture and the first word.

Participants were asked to look at the pictures and listen to the words. No behavioral response was required. The ERP experiment lasted approximately 15 minutes. The entire session was video recorded to make sure that children were looking at the visual displays for each trial. Six practice trials were added at the beginning, to familiarize the participants with the paradigm. For the duration of the experiment, the participants were seated in a comfortable chair positioned within an acoustically and electrically shielded chamber, while the EEG was recorded.

### EEG data acquisition

EEG data were continuously recorded from 64 scalp sites using an EGI recording system with a geodesic sensor net (Electric Geodesic, Inc., Eugene, Oregon, USA). The EEG signal was digitalized at a rate of 250 Hz and online bandpass filtered (0.1–100 Hz). As recommended for the EGI high input-impedance amplifier, electrode impedances were kept below 50 kΩ. The vertex sensor (electrode Cz) was used as the recording reference.

After recording, the EEG signals were bandpass filtered at 0.3–30 Hz in NetStation 3.0.2 and re-referenced offline to an average (whole head) reference. Eye movements were estimated from EEG at the electrodes slightly above and lateral to both eyes. Automatic EOG artifact correction was then applied (BESA research 5.2, Brain Electrical Source Analyses, BESA GmbH, Germany). Eye movement artifacts (e.g., blinks, saccades, and lateral movements) were corrected and removed using surrogate Multiple Source Eye Correction (MSEC) by Berg and Scherg [73] implemented within the BESA software. Channels with bad signal throughout the whole recording session (at most 20) were interpolated off-line using the BESA spline interpolation method. Of the 64 channels, an average of 16 channels (corresponding to 25%) were interpolated (19 for the ASD group and 14 for the Neurotypical group). The continuous EEG was segmented into epochs and categorized as to the match or mismatch between picture and word. For each trial, ERPs were time-locked to both the onset of the picture and the onset of the first word, in order to examine visual and auditory processing independently. In both cases, ERPs were calculated with respect to a pre-stimulus baseline of 100 ms for an epoch of 1500 ms for segments time-locked to the picture onset (used for analyses of basic visual processing) and for an epoch of 1000 ms for segments time-locked to the first word onset (used for analyses of basic auditory processing and lexical-semantic processing). The EEG segments were then subjected to an automatic rejection criterion applied to all electrodes. EEG Voltage thresholds were adjusted individually for each participant, ranging between 200 and 300 μV (neurotypical participants, *M* = 221, *SD* = 22.08, *min* = 200, *max* = 250; ASD participants, *M* = 268, *SD* = 29.27, *min* = 225, *max* = 300; *t*(18) = -4.054, *p* = .001). Overall, an average of 40 artifact-free EEG segments were used in each condition for averaging ERPs, with a minimum of 20 and a maximum of 50 trials for condition (neurotypical participants, Match condition, *M* = 44.9, *SD* = 7.5, *min* = 34, *max* = 49, Mismatch condition, *M* = 45.6, *SD* = 6.6, *min* = 35, *max* = 50; ASD participants, Match condition, *M* = 37.0, *SD* = 11.6, *min* = 20, *max* = 49, Mismatch condition, *M* = 35.2, *SD* = 11.0, *min* = 20, *max* = 50; Match condition, *t*(15.4) = 1.81, *p* = .090; Mismatch condition, *t*(14.8) = 2.56, *p* = .022).

### Analysis procedures

Separate analyses were conducted to assess early visual processing, auditory processing and higher level lexical-semantic processing in these NV/MV ASD children as compared to typically developing children.

*Global Field Power* (GFP) measures were calculated for each condition and for each participant. GFP reflects electrical field strength and is calculated as the standard deviation over all electrodes for each time sample [74]. The use of GFP minimizes observer bias because it uses all electrode channels [75]. Based on visual inspection of the GFP waveform, time windows were defined around local GFP maxima. The selected time windows were divided into smaller time intervals so that we could include time as a factor in our analyses. Mean amplitude in the selected time windows (TW) was submitted to repeated measures ANOVAs in order to test for reliable group differences in response strength, independent of spatial location.

To examine *topography*, data were first normalized for each participant by dividing the amplitude values at each site by the standard deviation across sites (i.e., GFP). We then used a spatial principal component analysis (PCA) using normalized data as the input, (thus, the input was effectively a correlational matrix). PCA is an effective tool for maximizing variance and is being more frequently used in analyses of multichannel ERPs [76]. The goal of this PCA was to select subsets of sites that could be collapsed (averaged) because they co-varied for a selected time interval (identified from the GFP). This strategy reduced the number of channels in our analyses and, at the same time, increased our signal-to-noise because noise across different channels would be reduced by averaging. Following the PCA, we selected the first four components from each analysis, which accounted for 95% to 99% of the variance for most analyses (reported below in the results). The four components (after normalizing component weights) were then submitted to a k-means cluster analysis to allow us to determine which sites were consistently grouped together across all four components. We examined solutions using a maximum of 14 clusters. Specifically, we could constrain the solution by the number of clusters (e.g., reducing the 65 electrode sites to 14 clusters). Note that this method circumvents the necessity of employing a rotation method because the same cluster solution will be arrived at whether the data are rotated or not. Our rationale for using the k-means cluster approach is that it is objective and allowed us to separate the data into different topographical regions (clusters) with no overlapping sites. The sites identified as belonging to the same cluster were then averaged. Clusters corresponding to target patterns (i.e., P1 peaks, PSW and N400) were selected for further analysis. The average amplitudes of the selected clusters were submitted to the ANOVAs, as described below.

### Statistical Analyses

#### Basic visual processing

For the first positive deflection (P1), mean amplitude was calculated for two 50ms-long TWs (150–200 and 200–250ms) while for the second positive deflection (PSW), mean amplitude was calculated for three such 50ms-long TWs (350–400, 400–450 and 450–500ms). These TWs were used to assess group difference in GFP and topography. Mixed-model ANOVAs were conducted for each peak (P1 and PSW), where the between-subject factor was *Group* (children with ASD vs. Neurotypical children) and the within-subject factors were *Condition* (Match vs. Mismatch) and *Time Window* (P1 TW: 150–200 vs. 200–250; PSW TW: 350–400 vs. 400–450 vs. 450–500). Even though congruency effects were not expected in these early time-windows (before Word 1 presentation), the two conditions were analyzed separately in order to assess test-retest reliability information and to confirm the stability of the peaks. Differences in topography were assessed using ANOVAs that included *Region*, as well as *Group*, *Condition* and *Time Window* as factors. The dependent measure for these analyses was the average amplitude of clusters (called arrays below) identified using the PCA/cluster analysis approach.

#### Basic auditory processing

Basic auditory processing was assessed using ERPs to Word 1 (W1). We did not include analyses for W2 because too few trials were retained after artifact reject for adequate signal/noise ratio. Baseline correction was 100 ms prior to the onset of the W1. Statistical analyses were conducted on the first positive deflection (Auditory P1). Mean amplitude was calculated in three, 20ms-long TWs (100–120, 120–140 and 140–160 ms) for the GFP measures and for the averaged clusters of channels (selected through PCA and K-means cluster analyses). Finer grained intervals for the auditory P1 were used because we expected more rapid changes for early auditory processing. Separate mixed-model ANOVAs were conducted for Auditory P1, where the between-subject factor was *Group* (children with ASD vs. Neurotypical children) and the within-subject factors were *Condition* (Match vs. Mismatch) and *Time Window* (100–120 vs. 120–140 vs. 140–160). Differences in topography were assessed using ANOVAs that included *Region*, as well as *Group*, *Condition* and *Time Window* as factors. The dependent measure for these analyses was the average amplitude of clusters (called “arrays” below) identified using the PCA/cluster analysis approach.

### Lexical-semantic processing

Lexical-semantic processing was assessed from Word 1 (W1). Baseline correction was 100 ms prior to the onset of the word. Mean amplitude was calculated in four, 150ms-long TWs (350–500, 500–650, 650–800 and 800–950 ms) for the GFP and topography. This wide time-window was used, consistent with previous studies on developmental population (see [7] for a review). Separate mixed-model ANOVAs were conducted for the N400 component, where the between-subject factor was *Group* (children with ASD vs. Neurotypical children) and the within-subject factors were *Condition* (Match vs. Mismatch) and *Time Window* (350–500 vs. 500–650 vs. 650–800 vs. 800–950). Analyses on the averaged clusters of channels were conducted on the amplitude of the ERP waveforms, specifically a 2x2x4 ANOVA, where the between-subject factor was *Group* (children with ASD vs. Neurotypical children) and the within-subject factor were *Condition* (Match vs. Mismatch) and *Time Window* (350–500 vs. 500–650 vs. 650–800 vs. 800–950).

Since the focus of the present work was on the similarities/differences between typically developing children and children with ASD, only significant results (*p* < .05) involving main effects of Group or interactions including the between-subject factor Group are reported.

## Results

### Basic visual processing

**Fig 2:**
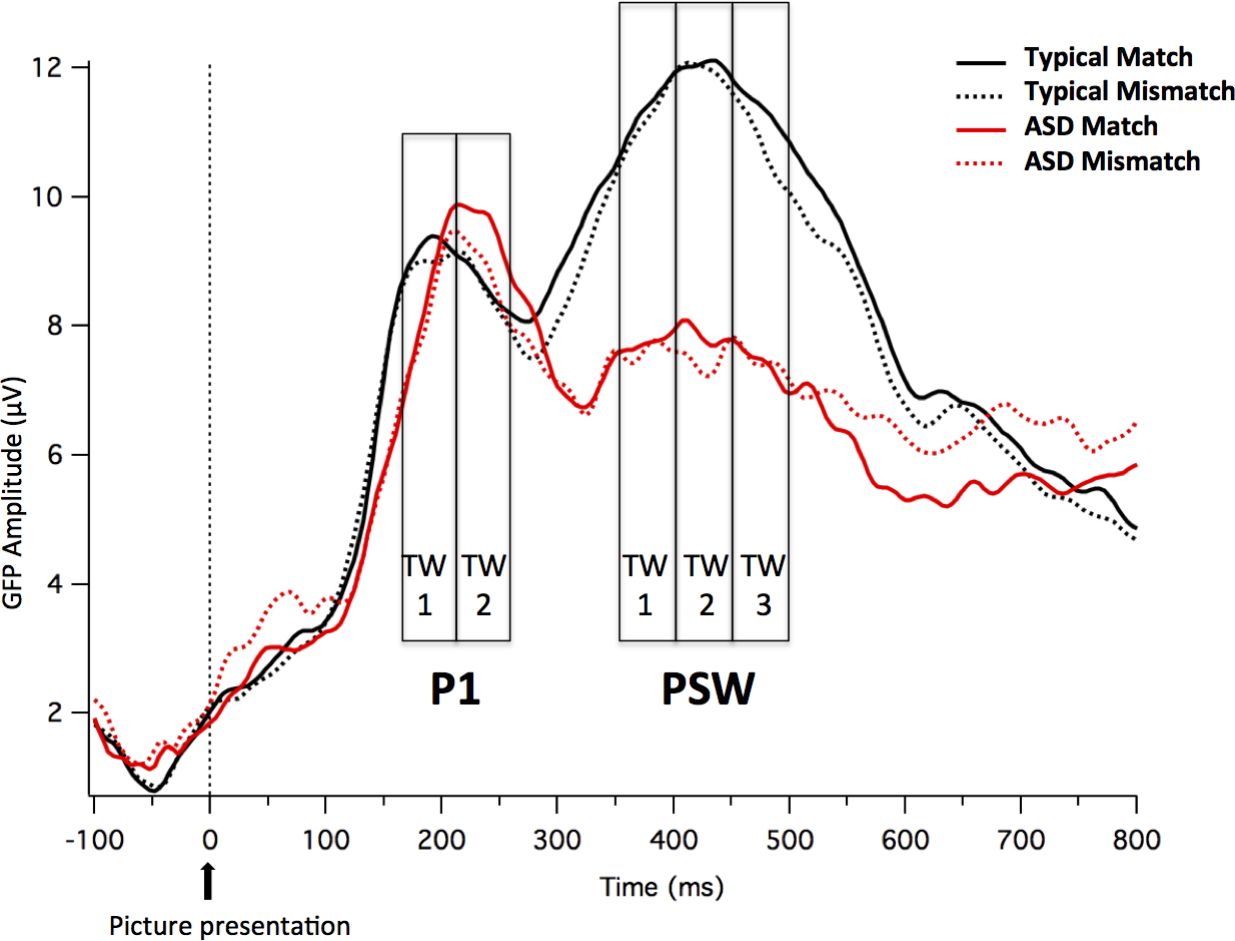
GFP of visual evoked potentials. GFP measures time-locked to the picture for the two groups (Neurotypical and ASD children) and the two conditions (Match and Mismatch).

In both groups, the visual stimuli elicited two positive peaks bifurcated by a negative-going peak. The first peak (P1) appeared at about 150–250 ms after picture onset. The second positive peak (PSW) appeared at about 350–500 ms (see Fig 2 for GFP of visual evoked potentials). Both peaks had the greatest amplitude over occipital sites.

#### Analyses of GFP for visual evoked potentials

**Fig 3:**
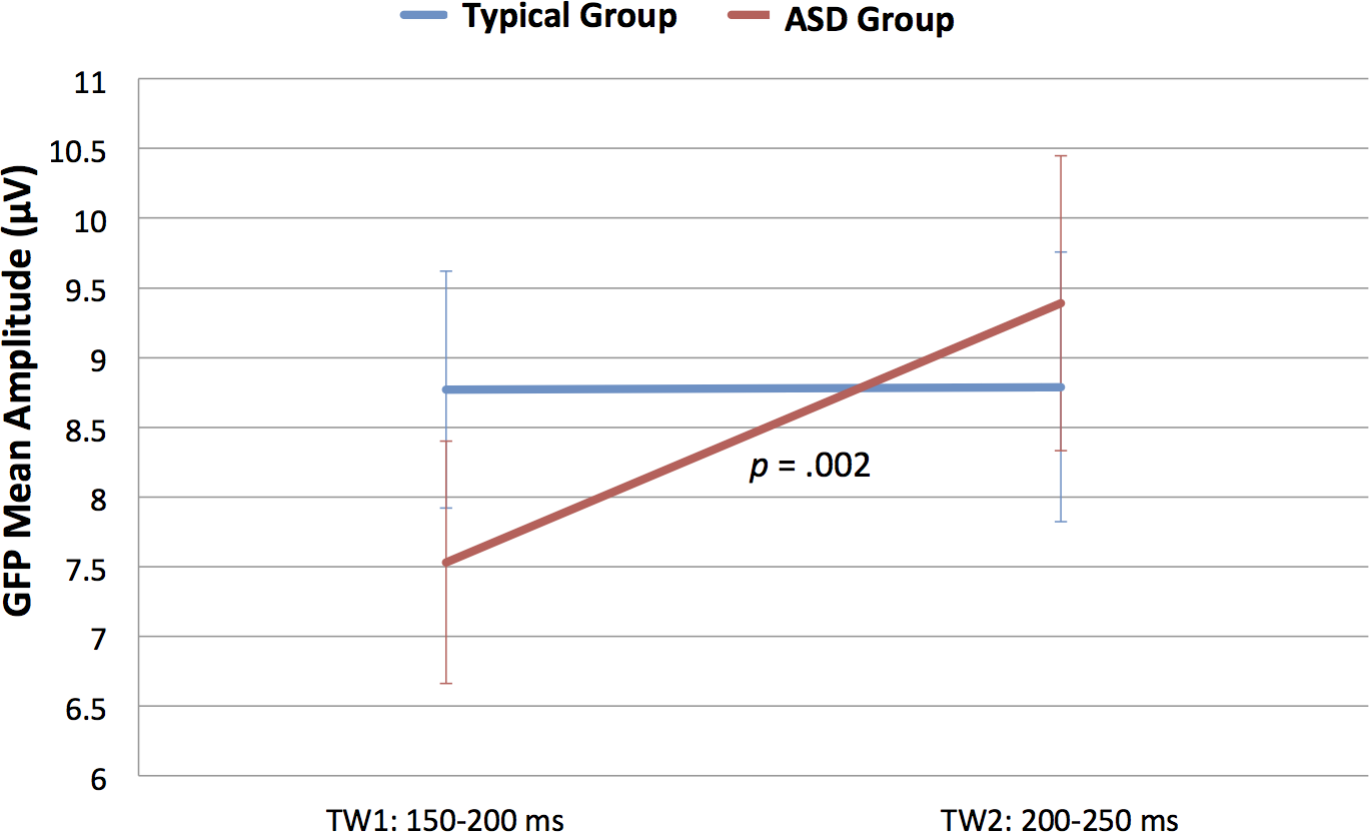
Time Window x Group interaction. Mean and standard error bars of the GFP to the picture (P1 peak) at the two time windows for the two groups.

Analysis of *mean amplitude of the GFP within the P1* TWs (150–200 and 200–250 ms) revealed a *Time Window x Group* interaction, *F*(1,18) = 4.489, *p* = .048, *ŋ*^*2*^ = .200. Fig 3 shows mean and standard error for GFP separately for Group and TW. Following up on this interaction, separate ANOVAs for groups revealed a main effect of *Time Window* in the ASD group, *F*(1,9) = 20.062, *p* = .002, *ŋ*^*2*^ = .690, but not in the Neurotypical group, *F*(1,9) = .001, *p* > .9. Children in the ASD group had greater P1 amplitude in later as compared to earlier TWs (TW 150–200: *M* = 7.53, *SD* = 0.87; TW 200–250: *M* = 9.39, *SD* = 1.06), whereas neurotypical children had comparable P1 amplitude in the two TWs (TW 150–200: *M* = 8.77, *SD* = 0.85; TW 200–250: *M* = 8.79, *SD* = 0.97).

Analysis of the *mean amplitude of GFP within the PSW* TWs (350–400, 400–450 and 450–500ms), revealed a main effect for *Group*, *F*(1,18) = 11.964, *p* = .003, *ŋ*^*2*^ = .399. The ASD group shows lower mean amplitude than the Neurotypical group (*M* = 7.45, *SD* = 2.33 and *M* = 10.88, *SD* = 2.74, respectively). No other significant main effects or interactions were found.

#### Topography: Visual PCA and cluster analysis for visual evoked potentials

**Fig 4:**
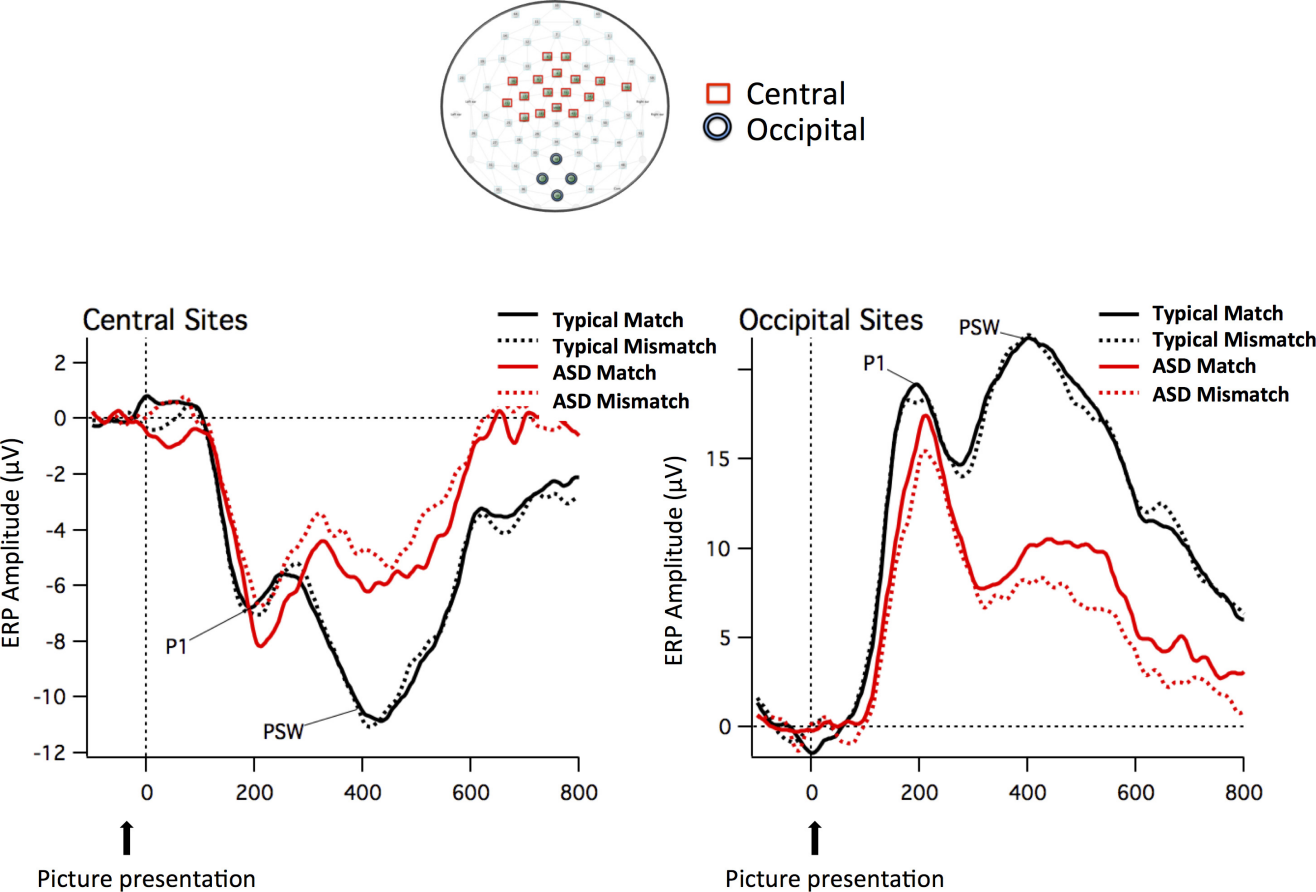
ERP waveforms of visual evoked potentials. ERP waveforms time-locked to the picture for the two groups (Neurotypical and ASD children) and the two conditions (Match and Mismatch) in the mean channels relative to the Central and Occipital sites included in the arrays. The sites that were used to create these arrays are displayed in the topographical map (Central sites in red and Occipital sites in green).

The PCA calculated on the normalized data (20 participants by two conditions) from 0 to 600 ms from picture onset revealed that four components accounted for 99% of the variance. The first component accounted for 93% of the variance and loaded most heavily on occipital site 40. Sites showing opposite polarity (and with PCA loadings with the opposite sign from occipital region sites) were largest near the vertex region (near site 55). Cluster analysis using the four components revealed that sites 37, 38, 39 and 40 were consistently clustered together. Around the vertex, central sites 3, 4, 5, 8, 9, 16, 17, 18, 21, 22, 43, 54, 55, 56, 57, 58 and 65 consistently clustered together. The sites in these clusters were averaged to create an occipital and a central array, which best represented the visual evoked potentials. Topographical maps included in Fig 4 display the sites that were used to create these mean values.

In order to explore topographic effects of the visual stimuli, the same ANOVAs were repeated on the averaged clusters, including the additional factor *Region* with two levels (occipital vs. central array). Analysis of the mean amplitude of the averaged clusters within the P1 TWs (150–200 and 200–250ms) uncovered a *Time Window x Region x Group* interaction *(F*(1,18) = 4.569, *p* = .047, *ŋ*^*2*^ = .202). Table 2 shows the Means and SDs for the mean amplitude in the occipital and central arrays, separately for Group and TW. To further examine the three-way interaction, the *Time Window x Region* interaction was analyzed separately by group and revealed a significant interaction in the ASD group, *F*(1,9) = 21.374, *p* = .001, *ŋ*^*2*^ = .704, but not in the Neurotypical group, *F*(1,9) = .000, *p* > .9. The greater P1 amplitude in the later as compared to earlier TW in the ASD group is thus evident for both the occipital and central arrays.

**Table 2:** Mean and standard deviations of the amplitude of the Occipital and Central arrays to the picture (P1 peak) at the two time windows for the two groups.

Group	Array	*TW1*: *150–200 ms*	*TW2*: *200–250 ms*	*t-test (p-value)*
		*M (SD)*	*M (SD)*	
**Neurotypical Group**	**Occipital**	17.63 (6.29)	17.40 (8.64)	.136 (.895)
	**Central**	-6.12 (2.97)	-6.34 (3.23)	.302 (.770)
**ASD Group**	**Occipital**	11.78 (6.65)	15.20 (7.90)	-3.559 **(.006)**
	**Central**	-4.77 (2.15)	-7.11 (3.23)	4.316 **(.002)**

Fig 4 shows the ERP waveforms time-locked to the picture for the two groups (Neurotypical and ASD children) and the two conditions (Match and Mismatch) in the mean channels relative to the Occipital and Central sites included in the arrays. S1 and S2 Figs show the ERP waveforms at the occipital and central sites (including both mean channels and the separate channels that co-vary based on the PCA analysis) by group and by condition.

Analysis of the mean amplitude of the averaged clusters within the PSW TWs (350–400, 400–450 and 450–500ms), revealed a main effect for *Group*, *F*(1,18) = 8.836, *p* = .008, *ŋ*^*2*^ = .329 and a *Region x Group* interaction, *F*(1,18) = 21.732, *p* < .001, *ŋ*^*2*^ = .547. In general, the ASD group exhibits lower mean amplitude than the Neurotypical group, reflected as less positive amplitude at occipital channels (ASD, *M* = 9.04, *SD* = 5.65; Neurotypical, *M* = 20.53, *SD* = 6.16; *t*(18) = 4.345, *p* < .001) and less negative amplitude at central channels (ASD, *M* = -4.96, *SD* = 2.92; Neurotypical, *M* = -9.97, *SD* = 2.81, respectively; *t*(18) = -3.904, *p* = .001).

### Basic auditory processing: Word 1

**Fig 5:**
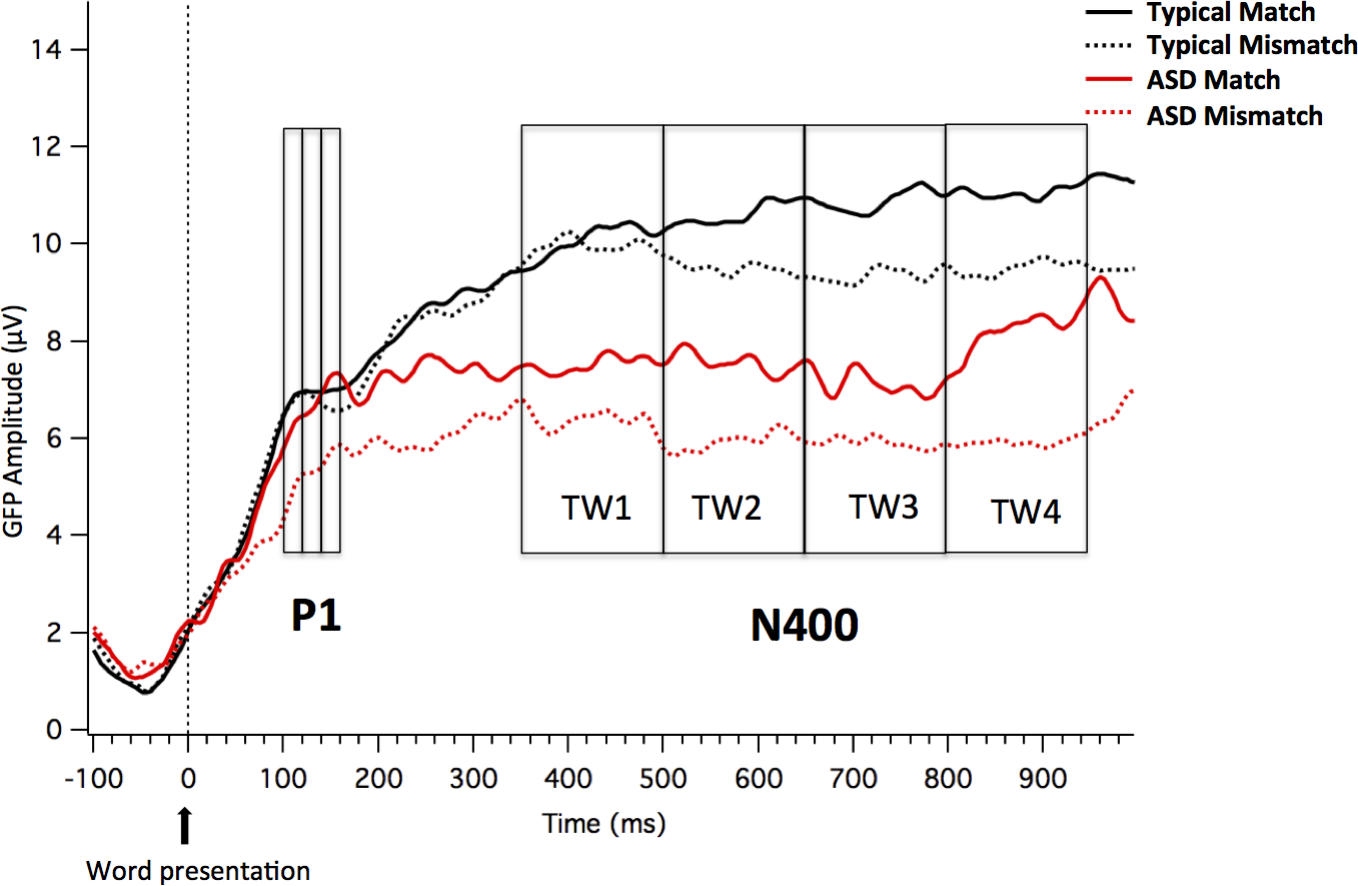
GFP for auditory evoked potentials. GFP measures time-locked to the word for the two groups (Neurotypical and ASD children) and the two conditions (Match and Mismatch).

In both groups, the auditory stimuli (Word 1) elicited a positive deflection at around 100–160 ms, identified as auditory P1 (see Fig 5).

#### Analyses of GFP for auditory evoked potentials

**Fig 6:**
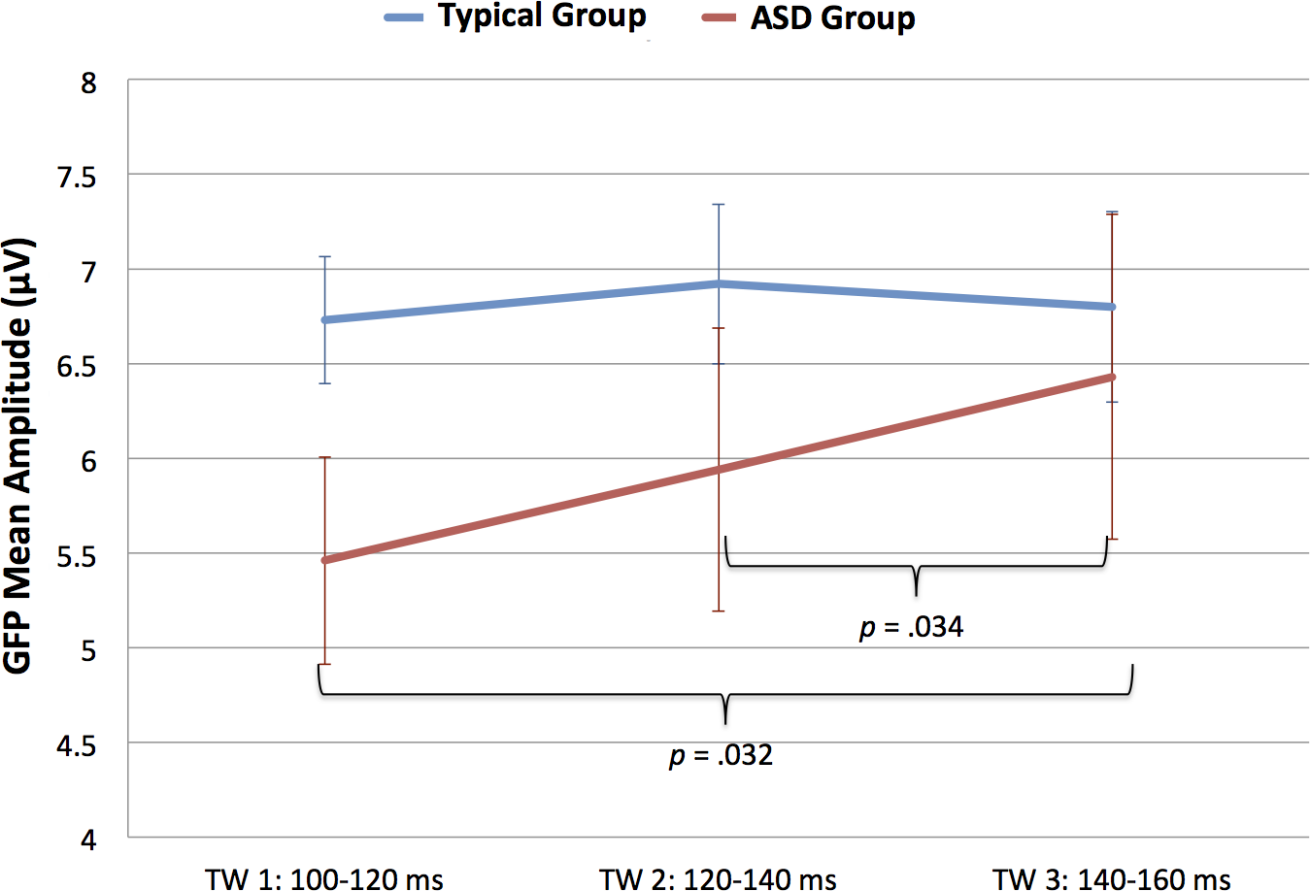
Time Window x Group interaction for auditory GFP. Mean and standard error bars of the GFP to the word (auditory P1 peak) at the three time windows for the two groups.

Analysis of GFP mean amplitude in the auditory P1 TWs (100–120, 120–140 and 140–160 ms) revealed a *Time Window x Group* interaction (*F*(2,36) = 3.528, *p* = .040, *ŋ*^*2*^ = .164). Fig 6 shows Mean and Standard Error for GFP separately by Group and TW. In order to explore the *Time Window x Group* interaction, the TW effect was analyzed separately by group. As expected, the main effect of *Time Window* was only significant for the ASD group, *(F*(2,18) = 5.844, *p* = .031, *ŋ*^*2*^ = .394), not for the Neurotypical group, *F*(2,18) = .507, *p* > .6. Post-hoc analysis suggests that children in the ASD group showed increased amplitude for the auditory P1 at the later TW (TW 140–160: *M* = 6.43, *SD* = 2.71), as compared to early TWs (TW 100–120: *M* = 5.47, *SD* = 1.73; TW 120–140: *M* = 5.94, *SD* = 2.36; significant results emerged when comparing mean amplitude between TW 100–120 and TW 140–160, *t*(9) = -2.538, *p* = .032, and mean amplitude between TW 120–140 and TW 140-160*t*(9) = -2.500, *p* = .034; see Fig 6).

#### Topography: Auditory PCA and cluster analysis

**Fig 7:**
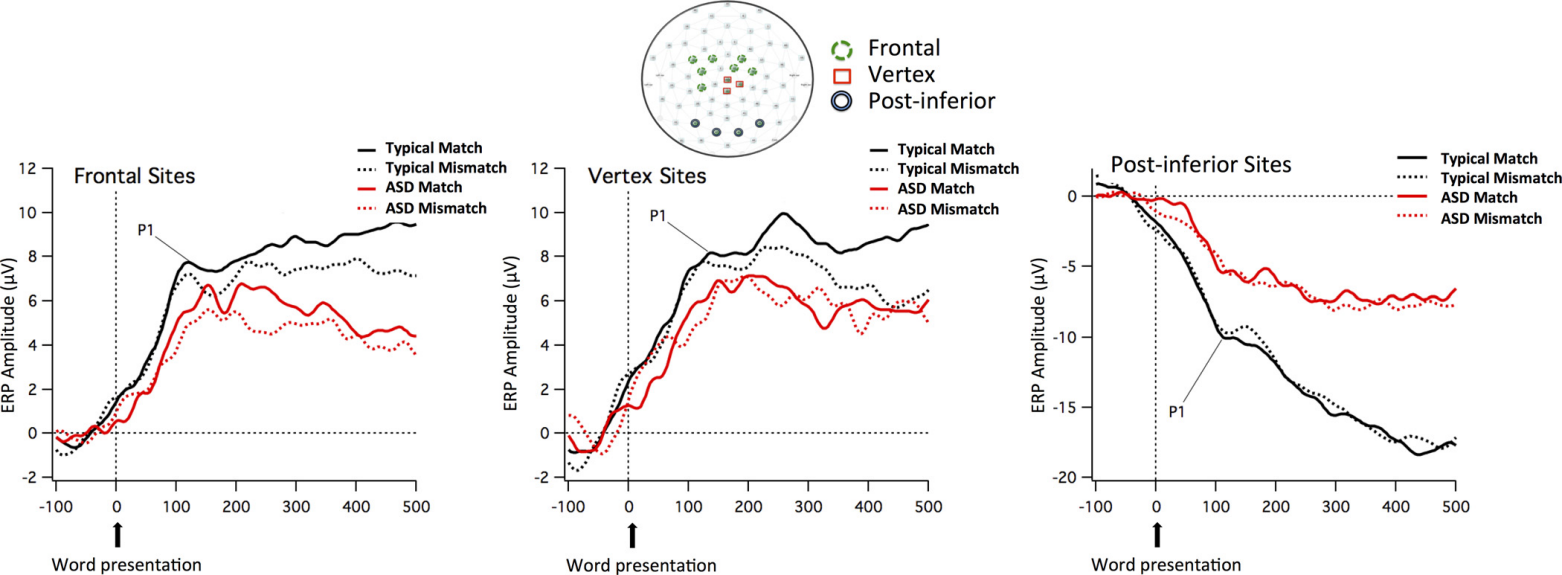
ERP waveforms of auditory evoked potentials. ERP waveforms time-locked to the word for the two groups (Neurotypical and ASD children) and the two conditions (Match and Mismatch) in the mean channels relative to the Frontal, Vertex, and Post-inferior sites included in the arrays. The sites that were used to create these arrays are displayed in the topographical map (Frontal sites in green, Vertex sites in red, and Post-inferior sites in black).

The PCA calculated on the normalized data (20 participants by two conditions) from 60 to 500 ms from word onset revealed that four components accounted for 99% of the variance. The first component explained 93.5% of the variance, with only 4.2% of the variance explained by the second component and less than 1% explained by a third component. Subsequent components explained overall less than 2% of the variance. The first component loaded most heavily on the vertex (and sites around the vertex) and on frontal sites and corresponded to a positive amplitude in the raw data. The loadings with an opposite sign were over posterior-inferior sites, which showed negative amplitudes at the peak in this time interval. Cluster analysis using the first four PCA components revealed that sites 30, 43 and Cz (around and at the vertex) were consistently grouped together. A second cluster was formed by frontal sites 9, 16, 17, 22, 54, 55, 58 and a third by post-inferior sites 32, 37, 40, 45. The sites in each of these three clusters were averaged to create vertex, frontal and post-inferior arrays, which best corresponded to the auditory P1. Topographical maps included in Fig 7 displays the sites that were used to create these mean values.

In order to explore topographic effects of the auditory stimuli, an ANOVA was performed with the addition of the within-subject factor *Region* (vertex vs. frontal vs. post-inferior array) to the factors of Time Window and Condition. Analysis of the mean amplitude of these arrays within the auditory P1 TWs (100–120, 120–140 and 200–250ms) uncovered a *Group x Region* interaction *(F*(2,36) = 6.841, *p* = .003, *ŋ*^*2*^ = .275). Irrespective of TWs or Conditions, neurotypical children had more positive amplitude than ASD children for the vertex array (Neurotypical, *M* = 7.56, *SD* = 2.28; ASD, *M* = 5.57, *SD* = 2.15; *t*(18) = 2.014, *p* = .059) and the frontal array (Neurotypical, *M* = 7.11, *SD* = 1.65; ASD, *M* = 5.12, *SD* = 2.51; *t*(18) = 2.096, *p* = .051) and more negative amplitude at post-inferior sites (Neurotypical, *M* = -9.57, *SD* = 4.00; ASD, *M* = -5.15, *SD* = 3.63; *t*(18) = -2.590, *p* = .018).

Fig 7 shows the ERP waveforms time-locked to the word for the two groups (Neurotypical and ASD children) and the two conditions (Match and Mismatch) for the mean channels relative to the vertex, frontal and post-inferior sites included in the clusters. S3–S5 Figs show the ERP waveforms at the frontal, vertex and post-inferior sites (including mean channels as well as separate channels that co-vary based on the PCA analysis) by group and condition.

### Lexical-semantic processing: Word 1

#### Analyses of GFP for later auditory evoked potentials

Analysis of GFP mean amplitude in only these late TWs (350–500, 500–650, 650–800 and 800–950ms) revealed a main effect of Group *F*(1,18) = 15.345, p = .001, *ŋ*^*2*^ = .460. The mean amplitude for the ASD group (*M* = 6.82, *SD* = 2.14) was lower than that for the Neurotypical group (*M* = 10.12, *SD* = 1.58). No other significant main effects or interactions were found.

#### Topography: PCA and cluster analysis for the Word Subtraction Waveforms

**Fig 8:**
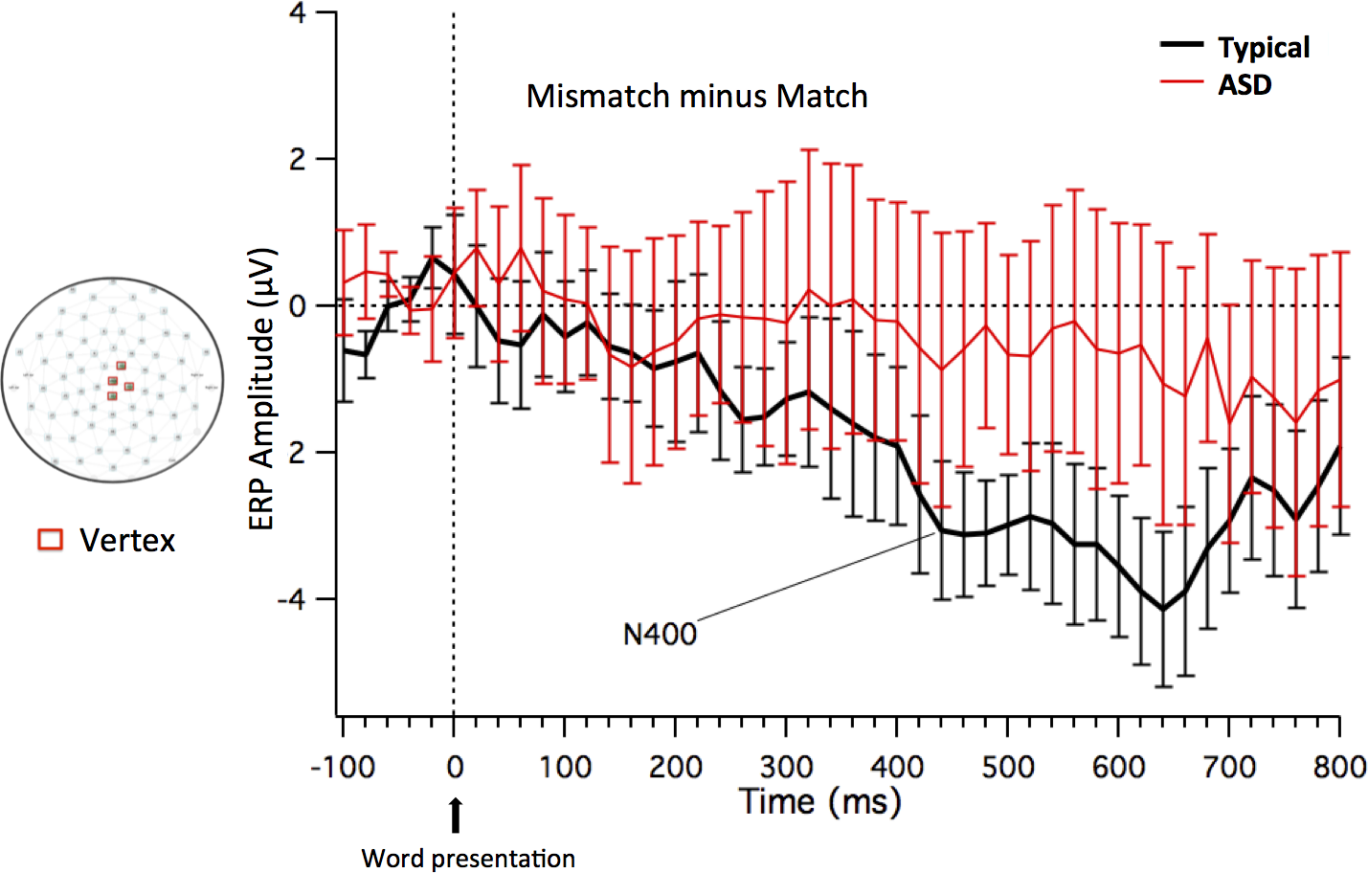
Word subtraction ERP waveforms. Subtraction ERP waveforms (Mismatch minus Match–including standard error bars) time-locked to Word 1 for the two groups (Neurotypical and ASD children) in the mean channel relative to the vertex sites included in the array. The sites that were used to create the Vertex array are displayed in the topographical map (in red).

The PCA was calculated on the normalized subtraction waves (20 participants by two conditions) from 400 to 800 ms from word onset revealed that the first four components accounted for 88% of the variance. The first component accounted for 52.1% of the variance, the second for 23.6% of the variance and the third for 8.3% of the variance; subsequent components explained, in total, around 15% of variance. The results of the cluster analysis revealed that the fronto-central sites at and around the vertex (sites 30, 43, 55, and Cz) were consistently grouped together. These sites showed relative negativity for the mismatch compared to the match condition, corresponding to the N400 effect. The sites in this cluster were averaged to create a vertex array (note that retaining 7 components in the PCA accounting for 95% of the variance leads to the same cluster result). Fig 8 shows the subtraction ERP waveforms (Mismatch minus Match), time-locked to the word for the two groups (Neurotypical and ASD children) in the mean channel relative to the vertex sites included in the array. The topographical maps included in Fig 8 display the sites that were used to create this mean value. S6 Fig shows the subtraction ERP waveforms at vertex sites (including both the mean channel and the separate channels that co-vary based on the PCA analysis) by group.

**Fig 9:**
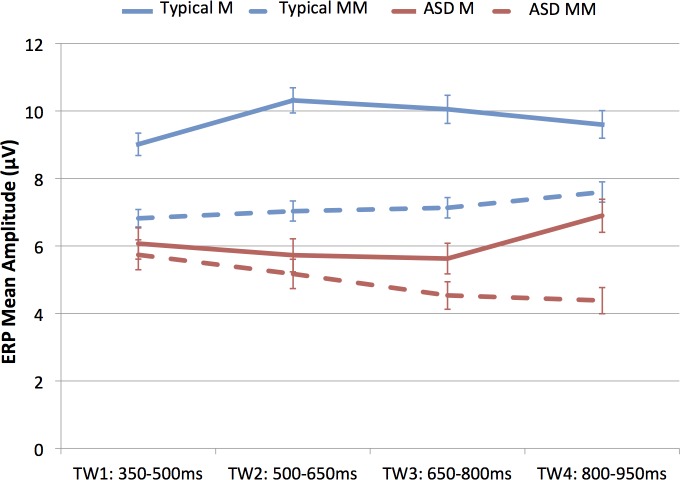
Group x Condition x Time Window interaction on the ERP waveforms time-locked to the word. Mean amplitude and standard error bars of ERP waveforms time-locked to the word for Match and Mismatch conditions (auditory N400 component) at the four time windows for the two groups.

A 2 x 2 x 4 ANOVA was conducted on the ERP waveforms for the vertex array. Analysis of the mean amplitude in the vertex cluster within the four TWs (350–500, 500–650, 650–800 and 800–950ms) uncovered a *Group x Condition x Time Window* interaction *(F*(3,54) = 3.109, *p* = .034, *ŋ*^*2*^ = .147) (see Fig 9).

To further examine the three-way interaction, the mean amplitude of the two conditions (Match vs. Mismatch) in the vertex array was compared by separate ANOVAs in each TW for each group separately. In the Neurotypical group, the two conditions differed significantly in all TWs except the last one, whereas in the ASD group, the two conditions did not differ in any of the TWs (see Table 3).

**Table 3:** Mean amplitude and standard deviations of the ERP waveforms in the two conditions (Match and Mismatch) time-locked to the auditory word for the vertex array at four time windows for Neurotypical and ASD groups.

TW	Neurotypical group			ASD group		
	Match	Mismatch	F (p-value)	Match	Mismatch	F (p-value)
	M (SD)	M (SD)		M (SD)	M (SD)	
**TW1: 350–500**	9.01 (3.32)	6.81 (2.58)	5.300 (**.047**)	6.07 (4.62)	5.73 (4.42)	0.039 (.847)
**TW1: 500–650**	10.31 (3.73)	7.03 (2.99)	14.013 (**.005**)	5.72 (4.91)	5.16 (4.33)	0.128 (.729)
**TW1: 650–800**	10.04 (4.18)	7.13 (3.03)	7.311 (**.024**)	5.62 (4.52)	4.53 (4.09)	0.435 (.526)
**TW1: 800–950**	9.59 (4.12)	7.59 (3.01)	4.359 (.066)	6.89 (4.91)	4.38 (3.88)	1.763 (.217)

### Individual patterns of ERPs

**Fig 10:**
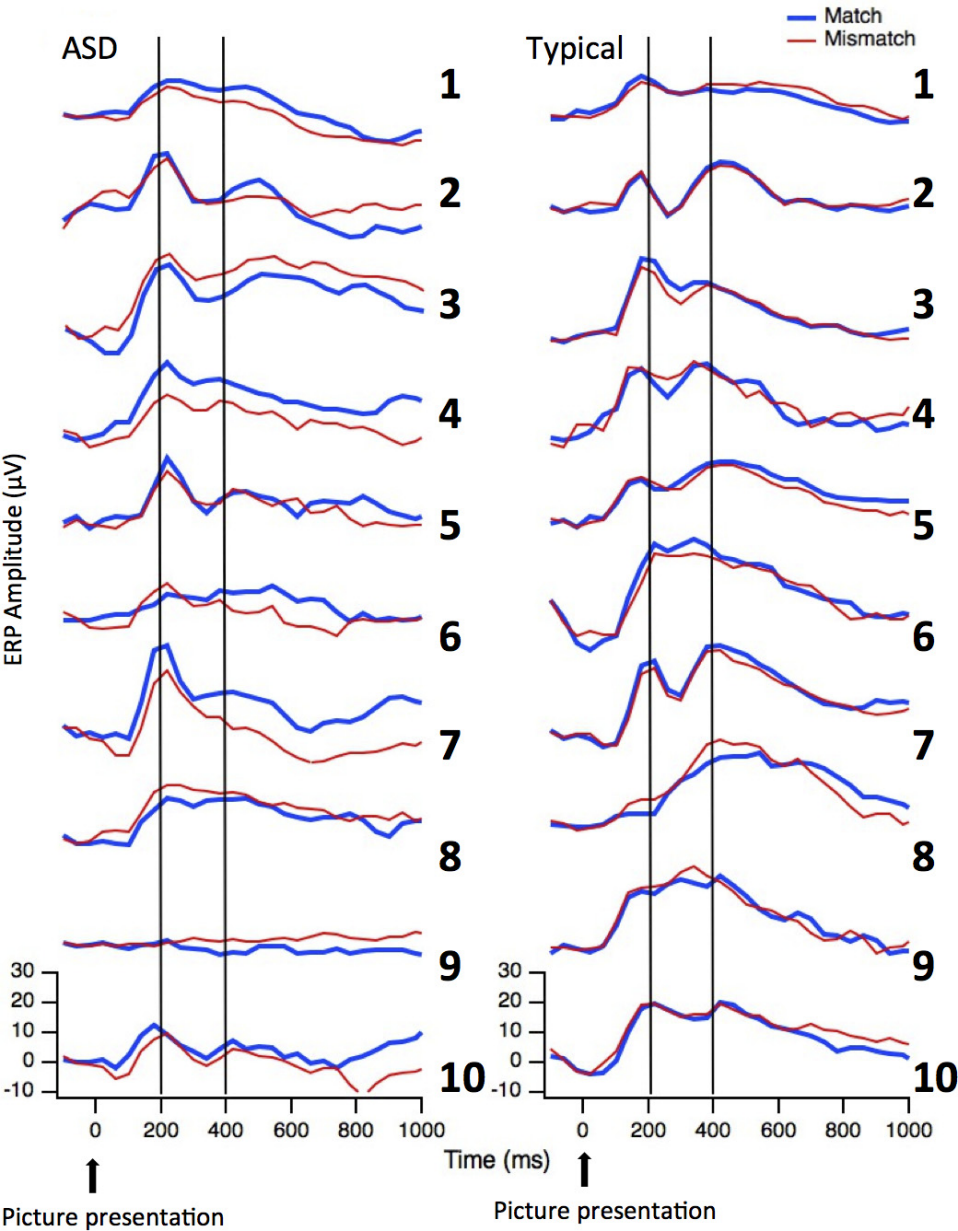
Individual ERP profiles for Visual Evoked Potentials. Individual ERP waveforms time-locked to the picture for the two groups (ASD children on the left and Neurotypical children on the right) and the two conditions (Match in blue and Mismatch in red) in the Occipital array. Vertical lines indicate the expected latency for the two peaks associated with basic visual processing: P1 and PSW.

**Fig 11:**
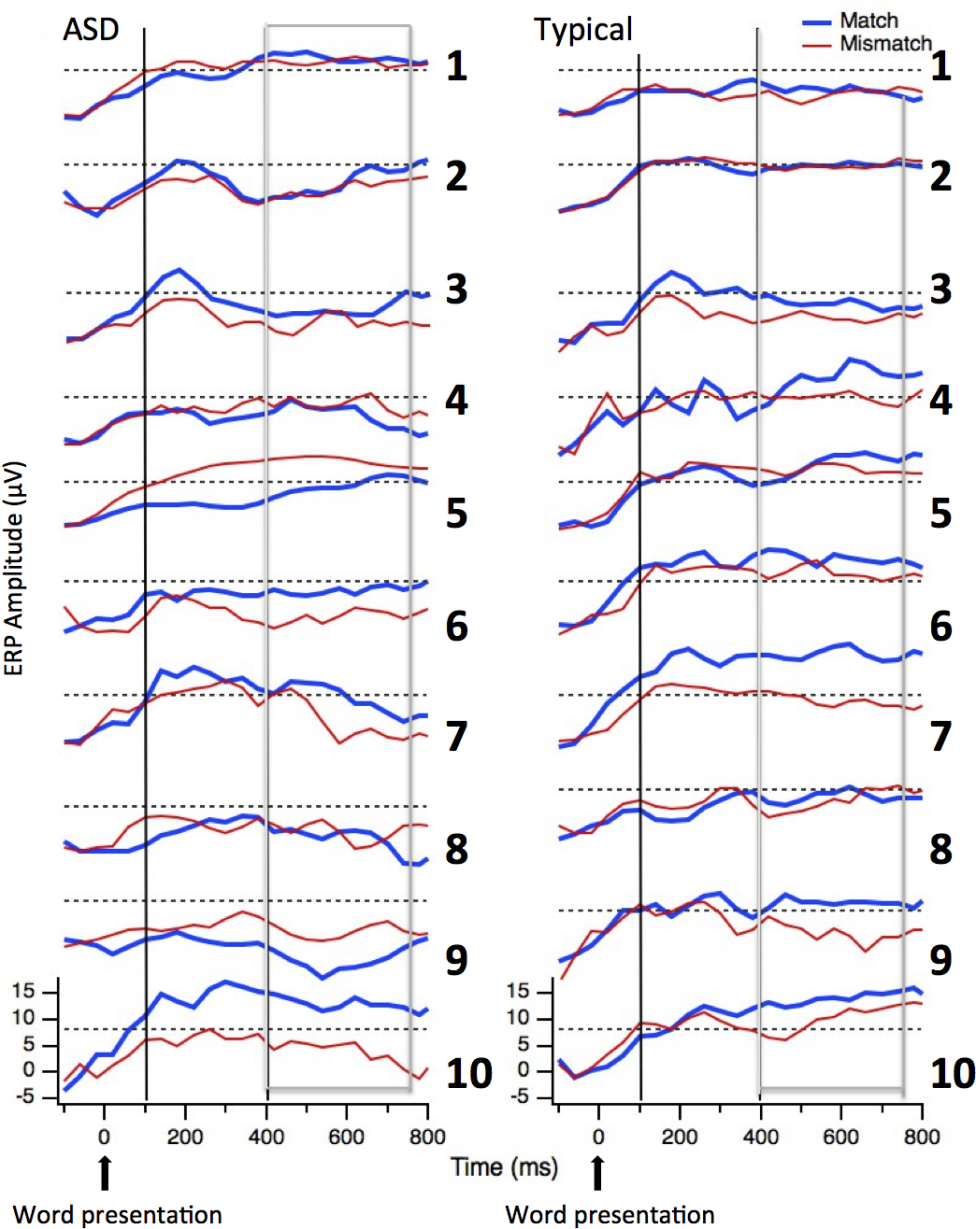
Individual ERP profiles for Auditory Evoked Potentials. Individual ERP waveforms time-locked to the word for the two groups (ASD children on the left and Neurotypical children on the right) and the two conditions (Match in blue and Mismatch in red) in the Vertex array. The vertical lines indicate the expected latency for the auditory P1 peak and the boxes indicate the time-window where the N400 component is expected.

Figs 10 and 11 show the individual ERPs time-locked respectively to the picture (Fig 10) and to the word (Fig 11) for both children in the ASD group and in the Neurotypical group. Table 4 summarizes the presence/absence of the different peaks/components at the individual level. As for the visual evoked potentials, only one child in each group did *not* show a clear P1 peak (subjects ASD9 and CNT8). Interestingly, and consistent with the group results, PSW was higher than P1 for eight neurotypical participants and for none of the ASD participants. As for the auditory evoked potentials, peaks at the individual levels were hard to identify in both groups.

**Table 4:** Summary of individual ERP profiles.

Group	ID	VEP P1	VEP PSW	VEP Correlation between conditions (M-MM)	AEP Correlation between conditions (M-MM)	N400
**ASD**	ASD1	**Y**	N	**0.97**	**0.88**	**Y**
	ASD2	**Y**	N	**0.89**	**0.95**	N
	ASD3	**Y**	N	**0.98**	**0.93**	**Y**
	ASD4	**Y**	N	**0.99**	**0.66**	N
	ASD5	**Y**	N	**0.95**	**0.82**	N
	ASD6	**Y**	N	0.77	**0.64**	**Y**
	ASD7	**Y**	N	**0.95**	**0.87**	**Y**
	ASD8	**Y**	N	**0.98**	0.45	N
	ASD9	N	N	-0.58	0.1	N
	ASD10	**Y**	N	**0.90**	**0.87**	**Y**
**NEUROTYPICAL**	TYP1	**Y**	N	**0.94**	0.48	**Y**
	TYP2	**Y**	**Y**	**0.98**	**0.96**	N
	TYP3	**Y**	N	**0.98**	**0.88**	**Y**
	TYP4	**Y**	**Y**	**0.94**	0.47	N
	TYP5	**Y**	**Y**	**0.97**	**0.96**	**Y**
	TYP6	**Y**	**Y**	**0.99**	**0.87**	**Y**
	TYP7	**Y**	**Y**	**0.99**	**0.94**	**Y**
	TYP8	N	**Y**	**0.97**	**0.64**	**Y**
	TYP9	**Y**	**Y**	**0.98**	**0.66**	**Y**
	TYP10	**Y**	**Y**	**0.99**	**0.78**	**Y**

Y = Yes, N = No, VEP = Visual Evoked Potentials, AEP = Auditory Evoked Potentials, PSW = Positive Slow Wave, M = Match between picture and auditory word, MM = mismatch between picture and auditory word

In addition to visual inspection, a correlation statistic for condition 1 (Match) compared to condition 2 (Mismatch) was performed in order to obtain test-retest reliability information to confirm the stability of the peaks. For each child, correlations were computed for the occipital sites for the visual evoked potentials and for the vertex sites for the auditory evoked potentials from 0 to 420 ms. As reported in Table 4, correlations are very high for both groups. For the visual evoked potentials at occipital sites correlations for eight ASD participants were above .89; only one of these children did not show a positive correlation, and in fact, conditions were negatively correlated (participant ASD9, r = -.58). All of the correlations for the neurotypical participants were above .94. For the auditory evoked potentials at Vertex sites, both groups showed lower correlations between the two conditions than for visual evoked potentials. This is not surprising, since the conditions differ in whether the word matched or mismatched the picture. Even so, the correlations were above .6 for eight of the participants in each group. Again the participant ASD9 is the only child without a positive correlation between the two conditions (r = .1) (see Table 4).

For the later auditory evoked potentials, presence/absence of a reliable N400 component at the individual level was estimated by means of t-tests between conditions (Match vs. Mismatch) considering all the time points in each TW. A subject was considered to show a reliable N400 if the t-tests were significant (at an alpha level of .05) in at least three TWs (with Mismatch more negative than Match). As reported in Table 4, five of the ASD children and eight of the neurotypical children showed a greater negativity to the Mismatch compared to Match condition.

## Discussion

The aims of this study were to assess basic perceptual processing of auditory and visual stimuli, as well as higher-order linguistic processing (lexical-semantic integration) in nonverbal (NV)/ minimally verbal (MV) children with Autism Spectrum Disorders (ASDs). The findings reported here are unique as this is the first study and part of a larger feasibility study, to assess perceptual and cognitive processing in this difficult-to-assess subset of ASD children using electrophysiology. Results of the ERP analyses are presented here and reveal significant differences between NV/MV ASD and typically-developing children, suggesting atypical brain activity in the ASD group at all levels of processing, but particularly at the lexical-semantic level (higher order process). In the following sections, we discuss the unusual pattern of electrocortical activity observed in this high-risk ASD population.

### Basic perceptual processing of visual and auditory stimuli

A clear pattern of differences emerged between NV/MV children with ASD and neurotypical children in processing both visual and auditory modalities. Analysis of the visual processing data revealed a number of commonalities as well as differences between the two groups. For example, both ASD and neurotypical children showed a two-peak response to the visual stimuli that was largest over occipital sites and inverted in polarity at fronto-central sites; however both peaks were found to differ between groups. Although the P1 peak did not differ between groups in amplitude, or in topography, it peaked in a later TW in the ASD group, suggesting longer latencies in ASD as compared to neurotypical children. This difference in the latency of the P1 peak suggests a general processing delay in the ASD group. The PSW (Positive Slow Wave) peak was reduced in amplitude in the ASD as compared to the Neurotypical group. Qualitative analyses performed at the individual level showed a clear P1 peak in 90% of the children in both groups. However, for 80% of the neurotypical children PSW was higher than P1, whereas none of the ASD participants had the same pattern. A functional interpretation of these findings suggests *relatively* intact early sensory processing abilities, as evidenced by the fact that only differences in latency and no differences in amplitude were found for the P1 peak. This is coupled with a divergence in processing mechanisms at the point when the visual responses are influenced in a top-down manner by rudimentary, even endogenously motivated, attention allocation processes. These findings corroborate previous findings suggesting that ASD individuals have atypical sensory processing abilities with regard to complex (but not simple) stimuli [31]. It should be noted that, as described in the introduction, the P1 peak is also mediated by attention, in the sense that its amplitude is larger when participants are attending to the stimuli [20–23]. There was no evidence that the two groups differed in allocation of attention to processing the pictures at this early point in processing, since there were no P1 amplitude differences, yet the underlying processing was delayed in the ASD group.

Functionally, the PSW has been described to be implicated in top-down, higher level processes associated with selective attention, response preparation, and/or the evaluation of response accuracy (see [77] for a review). In general terms, the PSW is described as a non-specific activation that signals the completion of any synchronized operation immediately following target detection [77]; PSW is seen as an index of the extent to which a stimulus is encoded and then updated in working memory [24]. This interpretation of the PSW seems at odds with the current study, where no explicit response was required of the children. However, we adopted this paradigm to determine whether there was evidence for the further visual processing that is critical for integration of the visual stimulus with the words that followed. The neurotypical children did indeed show evidence of further processing of the visual stimuli, whereas the high-risk NV/MV ASD children appeared to constrain their processing to the very basic visual characteristic of the pictures, without activating top-down mechanisms. Thus, the differences seen between groups at the PSW peak may reflect differences at the first step of semantic interpretation, suggesting that this is impaired in our NV/MV ASD cohort. Since the PSW in younger children is characterized by longer latencies (approximately 800 ms after stimulus onset [24, 78]), it is unlikely that the difference between groups found in our study (related to mean amplitude and not only to latency) could simply be related to maturational differences between the groups.

Analogous group differences emerged in auditory processing at the sensory level. As was seen for the visual P1 peak, similar amplitude levels were evident between groups for the auditory P1 peak. Moreover, the P1 showed similar topography in the two groups, with the largest amplitude over frontal-central sites, and inversion of polarity of inferior posterior sites. Despite this, the amplitude was greater for the ASD group in the later TWs, suggesting longer latencies in ASD as compared to the Neurotypical group. Overall, this again suggests *relatively* intact early sensory processing abilities, even in the auditory domain, although the significant difference in latency suggests a general processing delay for the ASD group. Interestingly, these results are comparable to those consistently found by Roberts and colleagues by means of magnetoencephalography (MEG), which reported latency delays (~10ms) of middle-latency M50/M100 responses in children with ASD as compared to those with typical development (see [79] for a recent review). In addition, a recent study conducted on cortical auditory evoked potentials in typically developing children by Shafer and colleagues [80] showed that latency shifts by about 20 ms from 3 to 7 years of age. Based on these results, the delay in latency for the ASD group in the auditory P1 could be interpreted as a maturational delay.

From the current findings, it is unknown to what extent the delays in sensory processing impact language acquisition and processing, but studies of children with specific language impairment–who clearly have problems at higher level of linguistic processing—have shown delays in information processing (e.g., [81]). Future studies will be needed to examine how speed of sensory processing is related to language acquisition in this population.

### Higher-order linguistic processing (lexical-semantic integration)

Finally, differences between neurotypical children and nonverbal/minimally verbal (NV/MV) children with ASD were most clearly seen at the level of lexical-semantic integration. In the neurotypical children, mismatched words elicited a clear N400 component between 350 and 800 ms after word onset (evident in the Mismatch minus Match subtraction waveforms). The developmental literature describes differences in the morphology and the topography of the N400 component in children as compared to adults [8,63–67]. Consistent with this literature, the N400 in our typically developing 4 to 8 year olds is characterized by later latency [65,67]. Children with ASD, as a group, did not exhibit any negativity, as evidenced statistically as well as on visual inspection of the grand average. The absence of statistical significance, even in the very late TWs (800–950 ms) might be due to the high variability in the sample of children with ASD. Visual inspection of the individual data revealed that five of 10 children with ASD showed a negativity that was consistent with the N400 effect, suggesting that a sub-sample of our ASD group *did* distinguish the two categories (matching and mismatching labels) and thus were able to process the stimuli at a higher linguistic level. However, no differences emerged at the behavioral level between children who show/do not show an N400 at the individual level. Only a few studies of children with ASD have examined semantic processing using an experimental design intended to elicit the N400. These studies have not observed an N400 modulation [50,51,68,69] even though these children had better-than-minimal language abilities. It is possible that sample variability contributed to this null finding. The study by Russo et al. [70], found evidence for semantic incongruence processing (pairs of pictures and sounds) in high-functioning adolescents with ASD, but at a different latency and by recruiting different areas of the cortex than neurotypical children. The McCleery et al. [69] study had a somewhat similar design to our study, but the children with ASD in their study showed better language skills. Partially in line with Russo et al.’s [70] results, they did observe an N400-like response to environmental sounds, suggesting that the children with ASD do access non-linguistic semantic information. However, they observed no N400 to words. It is possible that responses to the linguistic information in their study were more variable for children with ASD. This could be related to the choice of words and children’s individual experience with the words. Further investigation will be necessary to understand why McCleery et al. [69] did not find an N400. In particular, examination of individual results within both samples might be informative. However, for the present study, absence of an N400-like response for half of the ASD participants is less surprising, given that all our participants showed minimal language.

Taken together, our results suggest that the sample of NV/MV children with ASD in this study had *relatively* intact basic sensory processing skills which are comparable to those of typically-developing children, although generally with a delay in response time (for both visual and auditory stimuli). However, these children diverged from neurotypical children when higher-level processing was examined. Differences in the amplitude of the electrophysiological responses were found for both higher-level visual processing (e.g., more robust PSW component for the Neurotypical group), and for more sophisticated lexical-semantic processing, requiring visual-auditory integration (e.g., a significant N400 was only identified for the Neurotypical group). While the differences in latency seen in very early sensory processing suggest delays in initiating the underlying processes, nonetheless the responses appear comparable to those in typically-developing children. In particular, similar topography was observed for these early responses. However, the differences in amplitude found in the later, higher-level processing stages suggests the *absence* of these underlying process in at least a subset of the NV/MV children with ASD.

It is important to note that the evidence for atypical “higher-level” processing described here for our sample of NV/MV children with ASD does not necessarily mean that these children are *not* processing these linguistic stimuli. In fact, although children with ASD did not show an N400 effect at the group level, five of the 10 children did show greater negativity for the mismatched as compared to matched condition. It is possible that the children with ASD who did not show an N400 are indeed processing these stimuli at a higher cognitive level, but in a very different manner from neurotypical children, perhaps using compensatory strategies. Thus we cannot rule out the possibility that at least a subset of these NV/MV children may have more access to semantic information than is suggested from the group analyses. It is also important to keep in mind that a subset of the typically-developing children (two children) also failed to show a clear N400 effect. Further studies are needed to elucidate the nature of this heterogeneity. However, it is plausible that even in this highly compromised group, children who show a clear N400 effect may be more likely to develop verbal language skills with intervention than those who do not show such a pattern. Further exploration of these individual differences may facilitate identification of specific electrophysiological markers that could be used to predict language development in non-compliant populations such as these non-verbal or minimally-verbal children with ASD. At this point, visual inspection of the individual EEG/ERP data allows us to identify children who appear to be processing linguistic stimuli in a more typical way. Such qualitative analyses may pave the way to a more sophisticated child-by-child analysis, that may allow identification of those NV/MV children who may be the best candidates for specifically focused linguistic intervention and augmentative and alternative communication (AAC) technology.

### Neural Sources of the ERP Responses

Electrophysiology has excellent temporal resolution, but relatively poor spatial resolution due to the inverse problem (multiple source solutions can fit the data; [82]). Even so, inferences can be made regarding neural sources on the basis of topography on knowledge from other studies. The P1 and PSW responses to the picture were largest over the occipital region and showed inverse polarity at frontal-central sites. The more focal pattern over the occipital region (seen as fewer electrodes within a cluster) and a more diffuse pattern at frontal-central sites (seen as many electrodes in the cluster) are consistent with a source in occipital cortex. Thus, the PSW may at least partially reflect activation of occipital sites. The difference in overall power of the PSW (as reflected by GFP) for the two groups suggests that children with ASD either have fewer sources contributing to the response, or that they generally show less neural activation of the same sources as the neurotypical children.

The P1 response to the words showed the largest response over frontal-central sites and inverse polarity over inferior-posterior sites, consistent with a source in the superior temporal plane of auditory cortex [83]. Both groups of children showed this pattern and similar power for the P1 peak. This finding is consistent with intact neural sources in superior temporal cortex.

Sources contributing to the N400 are hypothesized to be more widespread [84]. Our finding of a more frontal topography of the N400 compared to studies with adults suggests that the contribution from underlying sources differs in young children. No studies to date have attempted to localize N400 in children, thus, at this time we can only speculate on which sources contribute to this response. We know from neuroimaging studies that the temporal pole, temporo-parietal juncture and angular gyrus all contribute to lexical access in adults [85,86]. Other prefrontal regions could also be activated in the process of selecting a lexical item. However, since we used a passive task, these areas are less likely to be strongly engaged. It is possible that the absence of a clear N400 response in a child who has sufficient lexical knowledge is due to maturational differences in how these different neural areas contribute to the scalp-recorded response. Future studies will be needed to explore this unresolved question, perhaps using a set of words in a setting that allows the child’s experience with each word to be assessed and then related to the neural responses.

### Limitations of the study

Limitations of the study include the small sample size for each group (n = 10), and thus lower power to detect small between-group differences. However, the differences we found between groups seem to be very stable and robust, and are consistent across the analyses of GFP and the channel arrays derived from PCA. Another critical issue and limitation of the present study is the relatively wide age-range of the sample (3 years, 7 months—7 years, 11 months). Developmental changes in the age range characterizing our sample are widely described in the literature, specifically for the obligatory auditory components [35,38,75]. The results relative to the auditory P1 might thus be those more affected by the developmental variability due to the relatively wide age-range of this sample. A further issue is that, overall, the children with ASD displayed noisier EEG data than neurotypical children. This is reflected in the need to set the thresholds for artifact rejection at a higher voltage for ASD children than for neurotypical children as well as the significantly lower number of trials remaining after artifact rejection for ASD children than for neurotypical children. Fewer trials may have led to greater noise even in the averaged data, since noise is reduced via averaging by the square root of the number of trials minus 1. To further minimize noise unrelated to the signal of interest, we averaged across sites that co-varied, as identified by our PCA and clustering method. It is not surprising that the children with ASD had noisier data and it is likely that this will remain a factor in future studies. Our study demonstrates the value of having data from multiple sites that allowed us to improve signal/noise ratios by averaging across sites measuring the same signal.

Finally, the fact that our children with ASD could not be tested using standardized IQ and language instruments limits our conclusions. This is an inherent obstacle when testing NV/MV children with ASD and a primary motivation for such studies. There are inevitably potential confounds to consider. First, differences in ERP responses between and within groups may be due to unmeasurable cognitive, linguistic and other abilities. Second, the N400 may be mitigated because the child does not know the target words. Further studies might include a pre-ERP lexical intervention that insures that the target words are familiar and have a strong baseline activation. A larger number of subjects would permit examining the extent to which other implicit measures of cognitive and language processing would predict the electrophysiological findings. Innovative and creative approaches to task design will be critical in order to move towards realistic and reliable assessment of linguistic and cognitive abilities in NV/MV ASD populations.

## Conclusions

The findings from this feasibility study suggest that passive EEG/ERP paradigms can provide useful and relevant information regarding sensory and lexical processing of NV/MV children with ASD, when standardized or adaptive testing cannot be used. Although it is difficult to prepare these children for such sessions (see [71]), and there may be limits to the types of tasks that can be administered (e.g., time limitations and the ability to examine stimulus properties passively), the information gained about language comprehension may far out-weigh the costs. This is particularly the case if the neural markers that appear to indicate a higher potential for development of language abilities prove to be predictive of later functional outcomes. The present study represents a first step in developing a functional neurolinguistic assessment for this extremely difficult-to-test population and could be the starting point for a rich body of work that challenges the notion that being nonverbal (that is “no spoken language”) implies an absence of language comprehension. In some individuals that may indeed be true, however, we have demonstrated that at least a subset of nonverbal ASD children may possess higher-level language processing skills. The challenge will be to optimize current ERP assessment paradigms and analysis protocols to provide sensitive, specific, and reliable measures that evaluate patterns of processing and semantic comprehension in these types of clinical populations. The next important step will be to link the responses observed in a subset of children with NV/MV ASD to functional outcomes.

## Supporting Information

S1 Appendix List of stimuli used in the picture-word matching paradigm. (DOCX)

S1 Fig ERP waveforms time-locked to the picture for the two groups (Neurotypical and ASD children) and the two conditions (Match and Mismatch) at the Occipital sites. The Figure includes both the average (Mean) and the separate channels included in the Occipital array.
(TIFF)

S2 Fig ERP waveforms time-locked to the picture for the two groups (Neurotypical and ASD children) and the two conditions (Match and Mismatch) at the Central sites. The Figure includes both the average (mean) and the separate channels included in the Central array.
(TIFF)

S3 Fig ERP waveforms time-locked to the word for the two groups (Neurotypical and ASD children) and the two conditions (Match and Mismatch) at the sites around the Vertex. The Figure includes both the average (Mean) and the separate channels included in the Vertex array.
(TIFF)

S4 Fig ERP waveforms time-locked to the word for the two groups (Neurotypical and ASD children) and the two conditions (Match and Mismatch) at the Frontal sites. The Figure includes both the average (Mean) and the separate channels included in the Frontal array.
(TIFF)

S5 Fig ERP waveforms time-locked to the word for the two groups (Neurotypical and ASD children) and the two conditions (Match and Mismatch) at the Post-inferior sites. The Figure includes both the average (Mean) and the separate channels included in the Post-inferior array.
(TIFF)

S6 Fig Subtraction ERP waveforms (Mismatch minus Match) time-locked to the word for the two groups (Neurotypical and ASD children) at the Vertex sites. The Figure includes both the average (Mean) and the separate channels included in the Vertex array.
(TIFF)

S1 Table Dataset used for statistical analysis. (XLSX)
