# Supplementary material for: From Sensory Perception to Lexical-Semantic Processing: An ERP Study in Non-Verbal Children with Autism
Source: PLoS One. 2016 Aug 25;11(8):e0161637. doi: 10.1371/journal.pone.0161637 (PMC4999236; doi:10.1371/journal.pone.0161637)
Supplement: S1 Appendix — (DOCX) [file pone.0161637.s001.docx]

**S1 Appendix. List of stimuli used in the picture-word matching paradigm.**

| **List of words matching the pictures:** | **List of mismatching words:** |
| --- | --- |
| Bed  Bee  Bike  Bird  Boat  Cake  Camel  Cat  Chicken  Dog  Drum  Duck  Fish  Frog  Hat  Key  Lion  Monkey  Mouse  Orange  Pencil  Phone  Pizza  Shoe  Squirrel  Sun  Train  Turtle  Watch  Zebra | Bottle  Bowl  Broom  Brush  Candle  Carrot  Chair  Cup  Desk  Doll  Fence  Flower  Fly  Glass  Guitar  Leaf  Moon  Pants  Pear  Pig  Plane  Sled  Spider  Spoon  Stove  Swing  Table  Tree  Truck  Wagon |
