# Supplementary figures and images for: From Sensory Perception to Lexical-Semantic Processing: An ERP Study in Non-Verbal Children with Autism
Source: PLoS One. 2016 Aug 25;11(8):e0161637. doi: 10.1371/journal.pone.0161637 (PMC4999236; doi:10.1371/journal.pone.0161637)

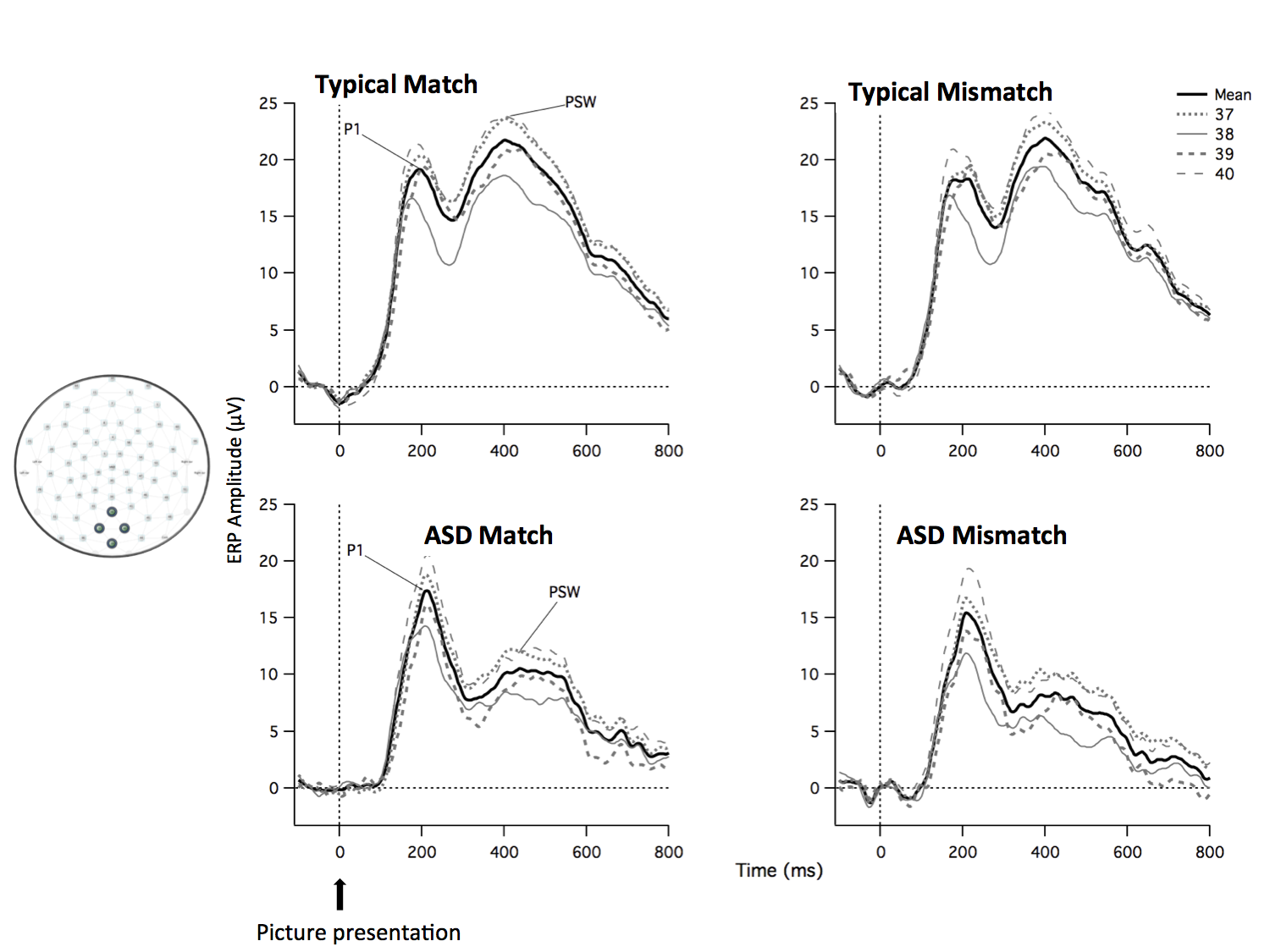

Supplement: S1 Fig — The Figure includes both the average (Mean) and the separate channels included in the Occipital array. (TIFF) [file pone.0161637.s002.tiff]

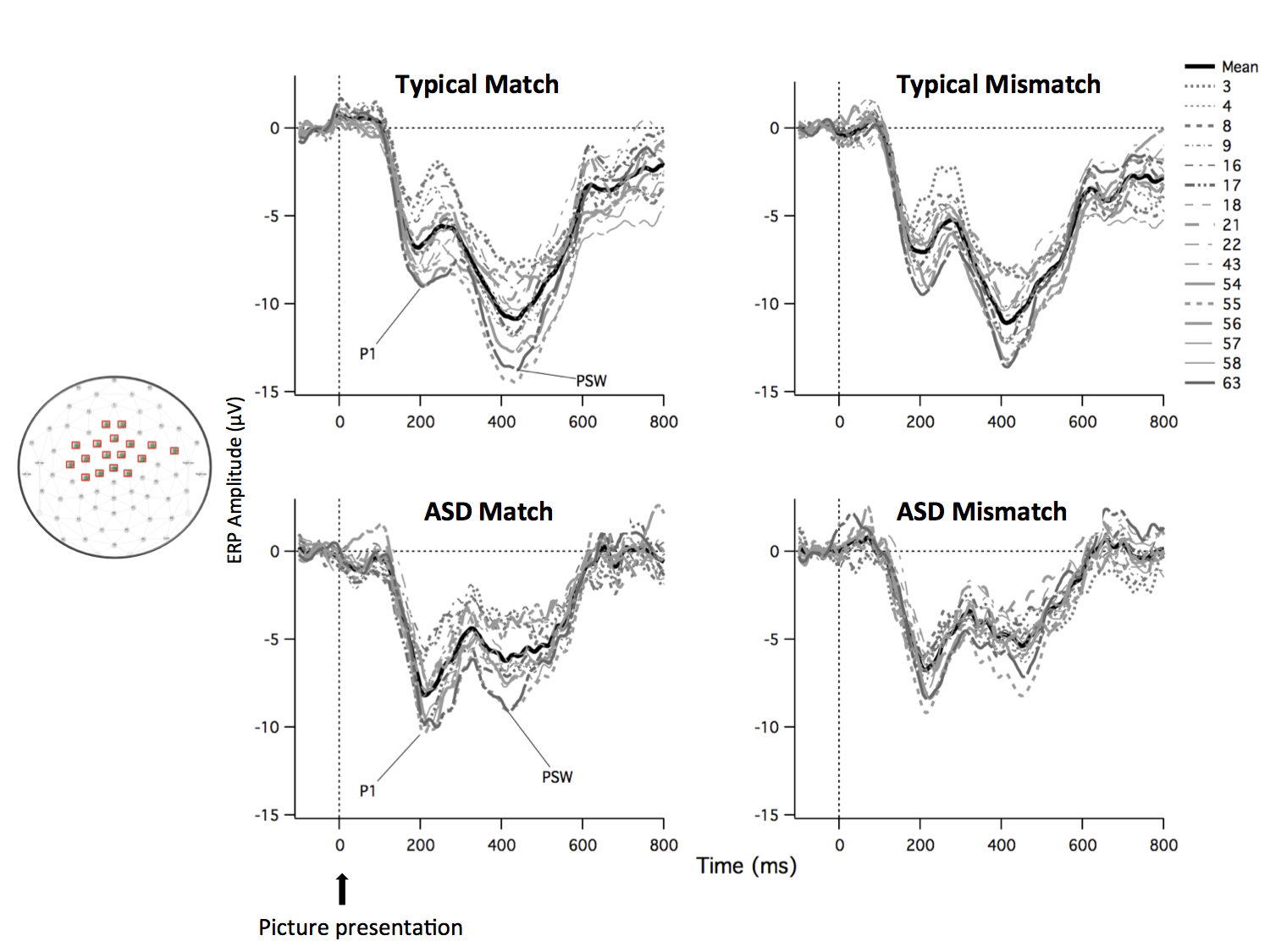

Supplement: S2 Fig — The Figure includes both the average (mean) and the separate channels included in the Central array. (TIFF) [file pone.0161637.s003.tiff]

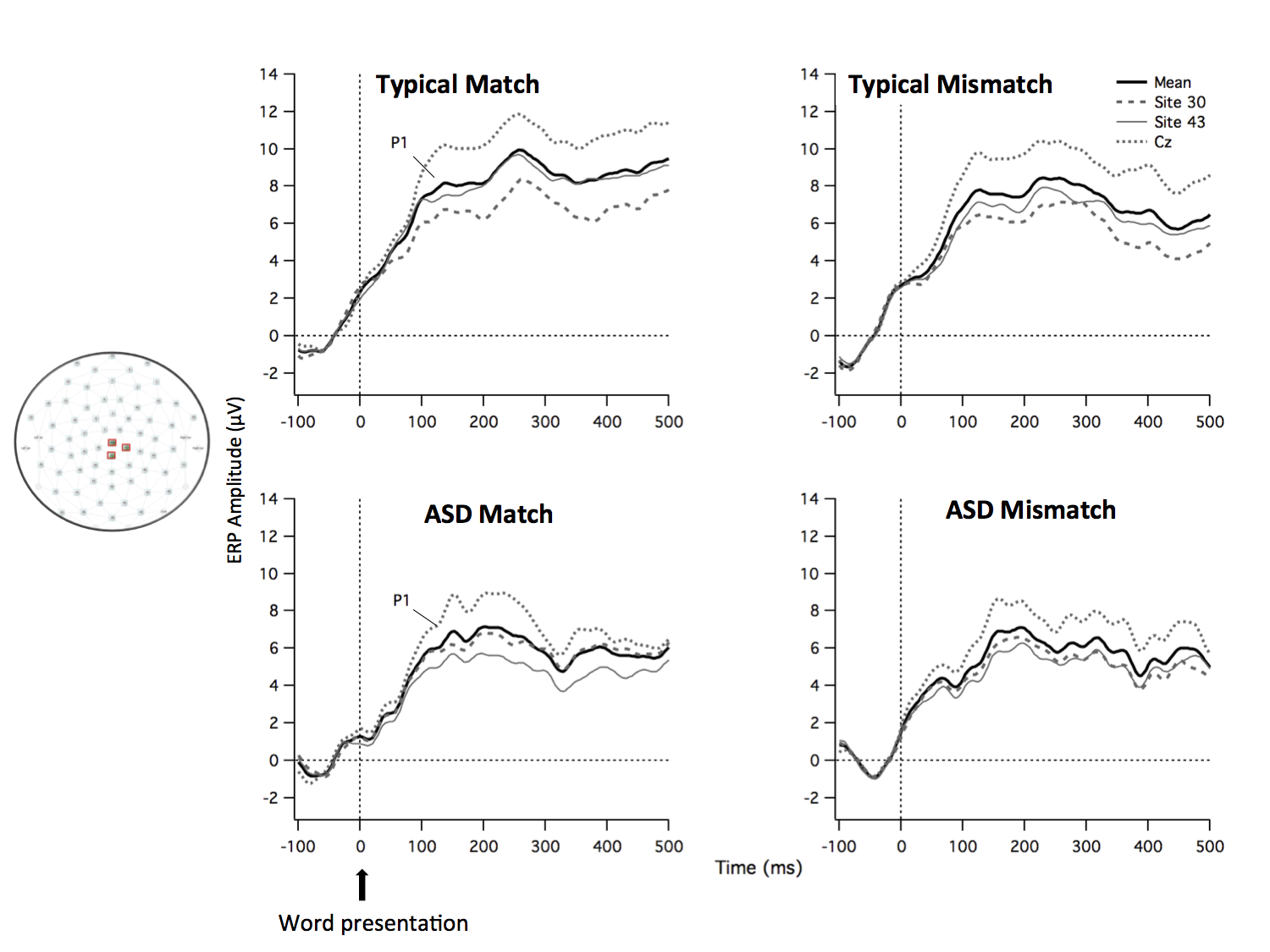

Supplement: S3 Fig — The Figure includes both the average (Mean) and the separate channels included in the Vertex array. (TIFF) [file pone.0161637.s004.tiff]

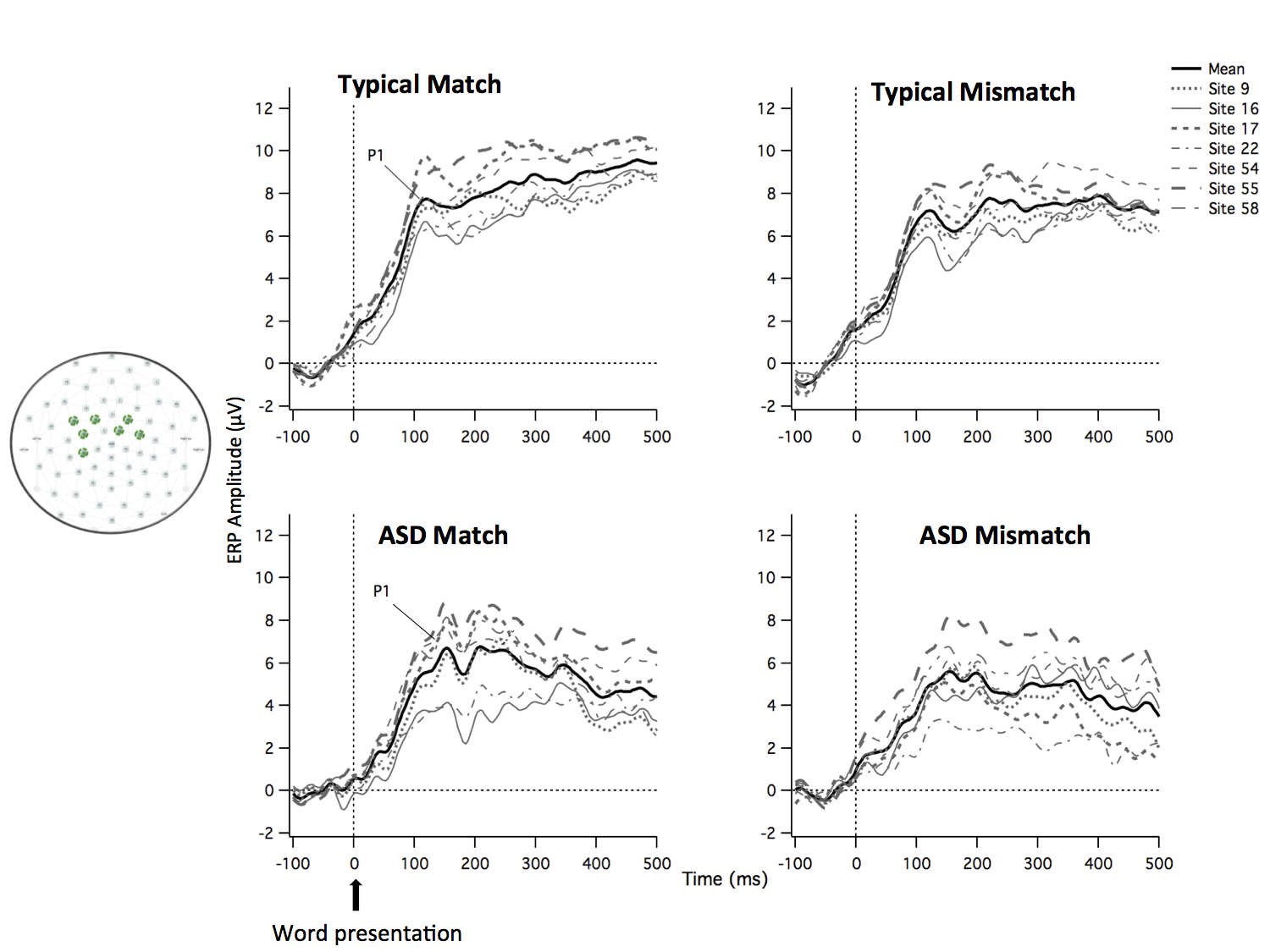

Supplement: S4 Fig — The Figure includes both the average (Mean) and the separate channels included in the Frontal array. (TIFF) [file pone.0161637.s005.tiff]

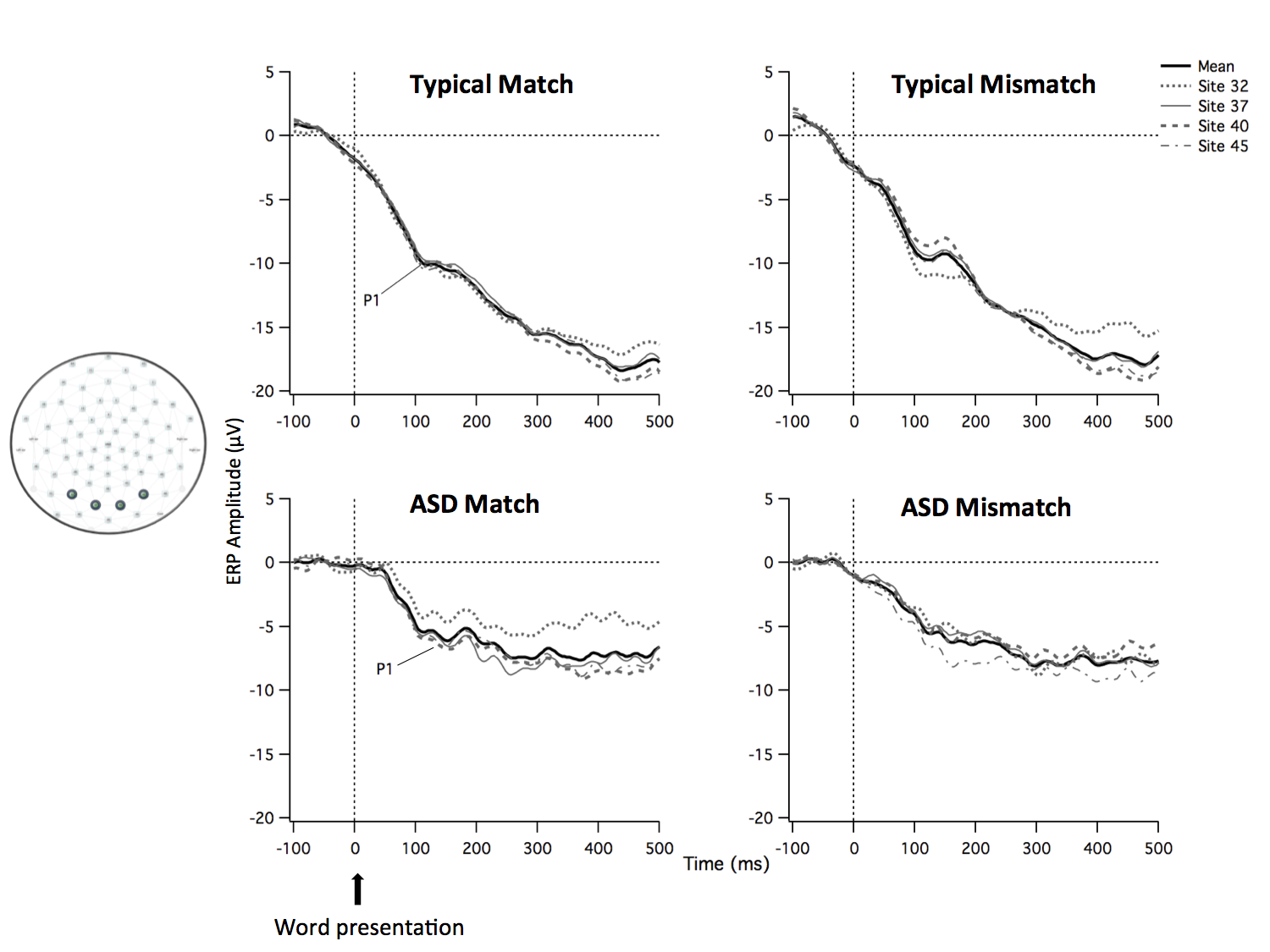

Supplement: S5 Fig — The Figure includes both the average (Mean) and the separate channels included in the Post-inferior array. (TIFF) [file pone.0161637.s006.tiff]

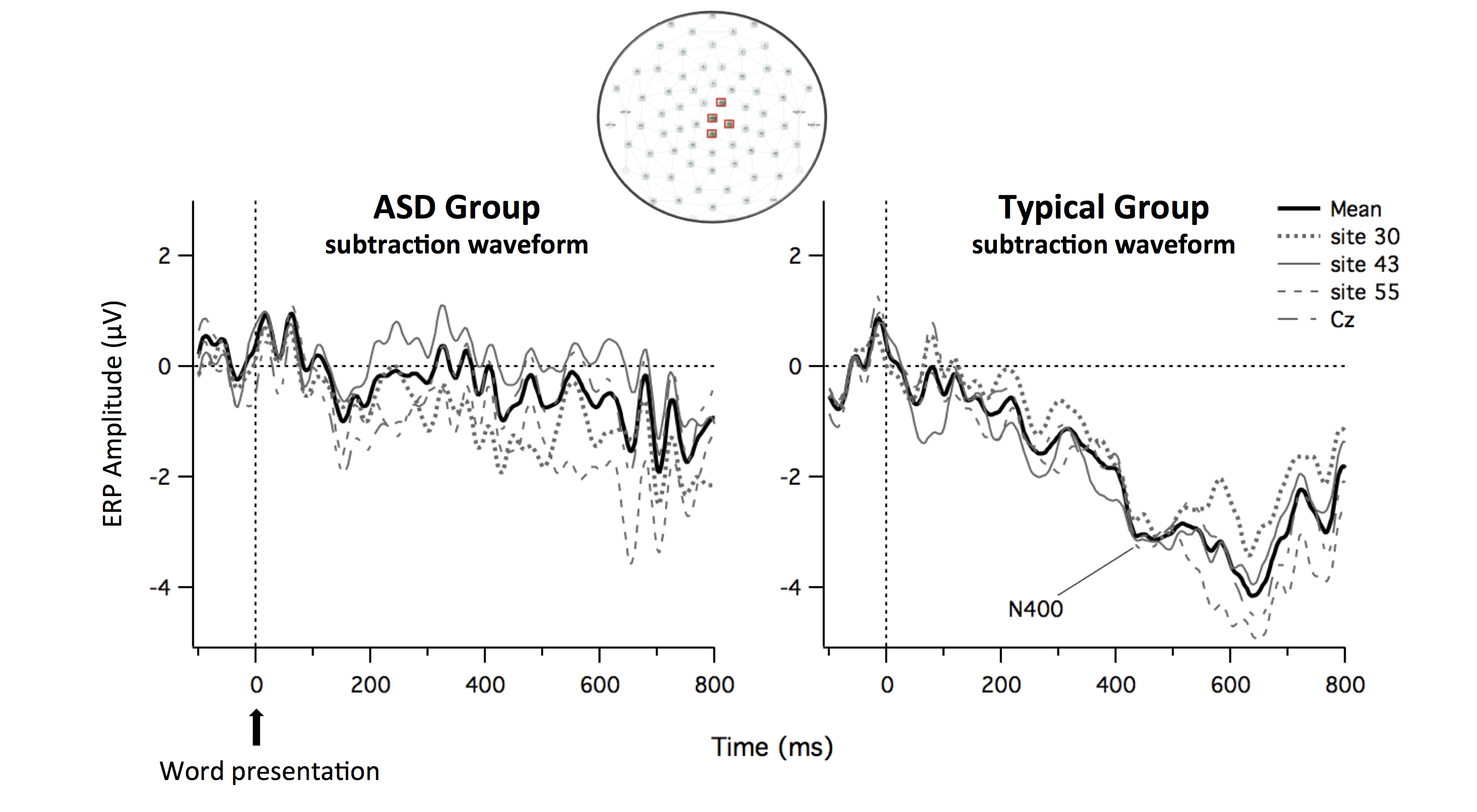

Supplement: S6 Fig — The Figure includes both the average (Mean) and the separate channels included in the Vertex array. (TIFF) [file pone.0161637.s007.tiff]
